# Divergent modes of episodic organization underlie whether emotional learning enhances memory across event boundaries

**DOI:** 10.3758/s13423-026-02924-5

**Published:** 2026-06-08

**Authors:** Blazej M. Baczkowski, Michiko Sakaki, Felix Kalbe, Kou Murayama, Lars Schwabe

**Affiliations:** 1 Hector Research Institute of Education Sciences and Psychology, University of Tübingen, Tübingen, Germany; 2 Research Institute, Kochi University of Technology, Kochi, Japan; 3 Department of Cognitive Psychology, University of Hamburg, Hamburg, Germany

**Keywords:** Episodic memory, Emotional learning, Threat conditioning, Event segmentation, Memory recognition

## Abstract

Episodic memory organizes continuous experience into discrete events, often limiting integration across temporal gaps. Emotionally salient experiences, such as learning about threats, may nevertheless enhance memory for motivationally relevant information across nominal event boundaries. Yet evidence for such cross-boundary modulation is mixed, suggesting that emotional learning may not exert a uniform effect on episodic memory across individuals. Here, we examined whether this heterogeneity reflects a mixture of divergent tendencies in how individuals structure their experience in memory – by temporal boundaries or by emotional relevance. Young adults (*N* = 285) incidentally encoded neutral images from two categories (animals, tools) across three temporally separated phases: pre-conditioning, conditioning, and post-conditioning. During conditioning, one category was partially reinforced with mild electric shocks to establish a category-level threat association. Memory was tested 24 h later using a surprise old-new recognition task. Although no reliable cross-phase enhancement was evident at the group level, data-driven individual-level analyses revealed two distinct patterns of episodic memory modulation. One pattern showed declining memory across phases, with emotional effects confined to shock-predictive items encoded during conditioning. The other showed memory structured around the emotional learning episode, with category-selective prioritization extending into the post-conditioning phase. These findings suggest that enhanced memory for subsequent threat-relevant information emerges specifically in individuals for whom the emotional learning episode, rather than nominal temporal structure, becomes the primary anchor of memory organization.

**Supplementary Information:**

The online version contains supplementary material available at 10.3758/s13423-026-02924-5.

## Introduction

Do we remember events primarily by when they occur, or by why they matter? Episodic memory actively transforms the continuous stream of experience into structured representations that support adaptive behavior (Nairne et al., 2007; Shohamy & Adcock, 2010). This requires selecting which moments belong together, which should be separated, and which should be prioritized for long-term storage. Temporal proximity typically provides a primary scaffold for grouping memories; however, experiences with high survival relevance can override this structure, linking distant moments and fragmenting contiguous ones (Clewett et al., 2019, 2025; Clewett & Davachi, 2017). Therefore, how we remember events reflects a fundamental tension between temporal continuity and behavioral relevance. Yet it remains unclear whether – and how – this organizational dynamic varies across individuals.

**Fig. 1:**
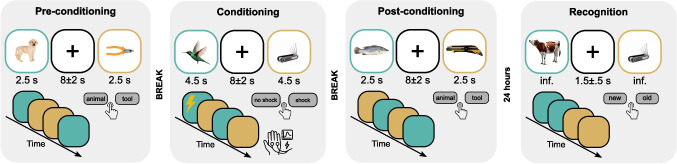
Experimental design. In each phase, participants incidentally encoded 30 unique images, each of animals and tools. During pre- and post-conditioning phases, they categorized images as animals or tools; in the conditioning phase, one category probabilistically predicted mild electric shocks, participants indicated their shock expectancy, and skin conductance responses were recorded as an index of autonomic arousal. About 24 h later, they completed a surprise old-new recognition test including all previously seen images and an equal number of novel category-level lures (confidence ratings not shown). Colored borders are illustrative. Visual elements adapted from flaticon.com (designed by flaticon, juicy_fish, Smashicons) and freepik.com (designed by freepik, evening_tao, brgfx)

The organization of experience in memory is strongly guided by event segmentation – the cognitive parsing of continuous experience into discrete episodes (Kurby & Zacks, 2008; Zacks & Swallow, 2007). Perceptual event boundaries, such as brief temporal gaps, impose constraints on memory by strengthening associations within the same segment while weakening associations across segments (Ezzyat & Davachi & Davachi, 2010; Ezzyat & Davachi, 2021). As a result, episodic memory tends to favor within-event coherence over integration across events, positioning temporal context as a default organizing principle (Dubrow & Davachi, 2016; Pu et al., 2022).

In parallel, episodic memory is shaped by internally evolving mental context – a representational state that drifts over time while integrating temporal, feature-based, and goal-related information (Howard & Kahana, 2002; Polyn et al., 2009). From this perspective, episodic structure depends not only on external perceptual boundaries but also on the continuity of internal representations (Chang et al., 2021; Manning, 2021). Factors that alter the rate or direction of internal context can therefore bias how experiences are grouped in memory, promoting either integration across time or segmentation into distinct episodes.

If episodic structure reflects an interaction between externally imposed boundaries and internally evolving context, emotionally salient experiences provide a particularly informative test case. Emotional events prioritize motivationally relevant information for long-term retention in episodic memory (Talmi, 2013; Williams et al., 2022), not only by strengthening item-specific encoding but also by stabilizing the internal contextual state (Clewett & McClay, 2024; Mcclay & Sachs, 2023). Under the account of segmentation by temporal gaps, the mnemonic influence of emotional events should be largely confined to information encoded during the emotional episode itself, with event boundaries limiting its impact on earlier or later neutral experiences. In contrast, if emotional salience alters contextual continuity by slowing contextual drift or maintaining an affectively biased contextual state across time (Lohnas & Howard, 2024; Talmi et al., 2019), its effects may extend beyond nominal event boundaries, biasing memory for neutral information encountered nearby in time (Tambini et al., 2016). Thus, the presence or absence of cross-boundary emotional memory effects provides a principled test case for examining whether episodic memory appears more strongly constrained by temporal structure or by relevance-weighted internal state.

Consistent with these competing accounts, empirical findings remain divided. Emotional learning – such as Pavlovian threat conditioning (Ledoux, 2000; Rescorla, 1988) – reliably enhances memory for information encoded during the emotional episode itself (de Voogd et al., 2016; Kalbe & Schwabe, 2022b). However, studies testing whether emotional learning extends beyond nominal event boundaries to affect threat-related and temporally adjacent information have yielded divergent results (Koevoet & Postma, 2023). While some report retroactive and proactive memory enhancement (Dunsmoor et al., 2015; Hennings et al., 2021; Laing et al., 2026), others find no reliable benefits beyond the emotional episode, even under close procedural replication (Kalbe & Schwabe, 2022a). Together, these findings suggest that cross-boundary mnemonic generalization is conditional, leaving open the question of when emotional learning enhances memory for both emotional and nearby neutral events.

Here, we propose that cross-boundary memory effects may not be uniform across individuals, but instead may depend on differences in how emotional learning interacts with episodic structure. Because episodic memory is governed by the interplay between temporal structure and relevance-weighted contextual processing, individuals may differ in how strongly they weight these principles. Consequently, null group-level effects may arise from averaging opposing tendencies: some individuals organize memory primarily according to temporal structure, whereas others prioritize emotional relevance (see also Bolger et al. (2019); Olsson-Collentine et al. (2020).

To examine this possibility, we reanalyzed the dataset reported by Kalbe and Schwabe (2022a). In this study, participants (*N* = 285) incidentally encoded neutral images from two semantic categories – animals and tools – across three sequential phases: pre-conditioning, conditioning, and post-conditioning (Fig. 1). Short breaks between phases served as nominal event boundaries. During conditioning, one category was partially reinforced with mild electric shocks, establishing a Pavlovian association at the category level. Consistent with prior work (Dunsmoor et al., 2012, 2014), conditioning enhanced 24-h recognition memory for items from the shock-predictive category encoded during aversive learning, irrespective of direct reinforcement. Yet, neutral items from the shock-predictive category encoded before or after conditioning showed no reliable group-level recognition memory advantage, making this dataset well suited to examine whether cross-boundary emotional effects vary systematically across individuals.

To capture individual-level variation in episodic organization, we applied a Bayesian latent mixture cognitive model of memory recognition (Lee & Stark, 2023; Nicenboim et al., 2025; Nicenboim & Vasishth, 2018). Rather than estimating overall memory strength or testing for isolated phase-specific effects of conditioning, this approach infers *latent memory profiles* defined as the structured pattern of memory performance across encoding phases and semantic categories. These profiles represent behavioral manifestations of underlying modes of episodic organization, each reflecting how the relative balance between temporal context and emotional relevance structures memory across encoding phases. One profile predicts selective enhancement for shock-predictive items encoded during conditioning, with no reliable category differences in the pre- or post-conditioning phases, consistent with memory organization governed primarily by nominal temporal boundaries. In contrast, an alternative profile predicts category-specific advantages that extend beyond conditioning, yielding retroactive or proactive enhancement for threat-related items, consistent with organization modulated by emotional significance. We therefore compared a single-profile model with a two-profile mixture model to determine whether memory performance reflects latent heterogeneity across individuals. Support for the mixture model would indicate that latent modes of episodic organization account for variation in whether emotional learning crosses event boundaries.

## Methods

### Data source

We re-analyzed data from Kalbe and Schwabe (2022a) who conducted four studies designed to replicate retroactive and proactive effects of Pavlovian threat conditioning on episodic memory. All studies closely followed the procedure of Dunsmoor et al. (2015) with minor deviations.

Studies 1–3 introduced two modifications: slightly altered stimulus-shock timing in delay conditioning and random (rather than typicality-balanced) assignment of stimuli to encoding phases. Study 1 also used a different but conceptually similar stimulus set (animals and tools with different exemplars) and collected old/new judgments separately from confidence ratings. Study 3 additionally extended the interval between preconditioning and conditioning from ~10 to 20 min. Study 4, which was pre-reviewed and pre-registered before data collection (https://osf.io/9hzmk), directly replicated the original procedure, matching stimulus-shock timing and balancing stimulus typicality across encoding phases.

Behavioral data were obtained from a public repository (https://osf.io/qpm3t), with additional demographic and skin conductance data provided by the corresponding author under a CC-BY 4.0 license.

### Participants

Data from all available participants (*N* = 285) were analyzed. Demographic data were available for 277 participants (191 female), aged 18–35 years (mode = 26). Skin conductance data were available for 272 participants (185 female), all within the same age range.

### Data collection procedures

The original protocol was approved by the local ethics committee. All participants gave written informed consent and were compensated. The present re-analysis required no additional ethical approval. Full methods are reported in Kalbe and Schwabe (2022a).

Briefly, each study comprised two sessions ~24 h apart. In session 1, participants incidentally encoded 180 unique neutral images from two semantic categories (animals and tools) across three separate phases: pre-conditioning, conditioning, and post-conditioning. In pre- and post-conditioning, participants categorized 60 images per phase (30 per category), presented for 2.5 s with inter-stimulus intervals of 6 ± 2 s.

During conditioning, participants viewed 60 additional images (30 per category), each presented for 4.5 s followed by an 8 ± 2 s fixation, while electrodes measuring skin conductance and delivering electric shocks were attached. Images from one category (CS+) predicted shock (US) at a 2/3 contingency, while images from the other category (CS–) were never paired. Category assignment to CS+ and CS– was pseudo-randomized across participants. On each trial, participants indicated whether they expected a shock (yes vs. no), knowing their responses would not influence outcomes.

In session 2, participants completed a surprise old-new recognition test with 360 images, in which old images were mixed with an equal number of new images from each semantic category.

### Measures

#### Old-new recognition judgments

In Study 1, recognition judgments were collected in two stages: an initial old/new decision followed by a four-point confidence rating (*very unsure*, *rather unsure*, *rather sure*, and *very sure*), with a 5-s response window. Consequently, trial counts varied slightly across participants, with 12 out of 44 participants missing only a few trials (mode = 1). In the other three studies, participants selected one of four combined memory-and-confidence responses without a time limit: *definitely old*, *maybe old*, *maybe new*, or *definitely new*.

### Data analysis

Before fitting any models, we conducted data-quality and reproducibility checks. We visually inspected hits and false alarms (FA) and reproduced key analyses from Kalbe & Schwabe (2022a), including overall performance, repeated-measures ANOVA, and paired t-tests on corrected recognition scores (hit rate - FA rate; see the Supplement).

After confirming data integrity, we conducted four analysis steps to quantify how emotional learning influences episodic memory across nominal event boundaries. First, we applied the standard two-high-threshold (2HT) cognitive model to old-new judgments to distinguish mnemonic from non-mnemonic processes underlying responses in the surprise recognition test (Snodgrass & Corwin, 1988). Second, we estimated model parameters within a hierarchical Bayesian framework – implemented as a generalized (logistic) linear mixed model – to obtain stable participant-level estimates while allowing individual variability. Third, we explored individual differences in memory-based recognition performance using dimensionality reduction and clustering of participant-level parameters. This step provided a descriptive view of potential subgroup structure without imposing categorical assumptions. Finally, we formally tested whether inconsistent group-level effects reflect latent heterogeneity in episodic organization by extending the model with a mixture component capturing two memory profiles: one reflecting temporal-context-based organization (emotional enhancement restricted to the conditioning phase due to nominal boundaries), and the other reflecting emotional-relevance-based organization (enhanced memory for items from the shock-predictive category encoded before and after conditioning).

#### Two-high-threshold (2HT) recognition memory process model

To assess individual heterogeneity in how emotional learning shapes episodic memory, we used a cognitive process model that separates mnemonic from non-mnemonic influences on recognition judgments. Specifically, we applied the 2HT recognition memory model, which explains old-new decisions in terms of two mutually exclusive latent cognitive states: memory-based detection and guessing (Snodgrass & Corwin, 1988). These latent quantities cannot be recovered from summary measures and are necessary to examine individual differences in memory processes, rather than overall response rates. This process-level framework also aligns theoretically with prior work using the same experimental design (Dunsmoor et al., 2015).

In this design, three phase-specific target conditions and a single lure condition introduce a target-lure asymmetry that violates the assumptions under which simpler metrics – such as the corrected recognition (CR) score derived from 2HT model – reflect memory-based detection (see Supplementary Appendix A). CR also collapses mnemonic and non-mnemonic processes into a single value, limiting the analysis of individual differences (i.e., identical CR can arise from different detection-guessing combinations). Therefore, we analyzed the recognition data using the full generative 2HT formulation, which accommodates the target-lure asymmetry by treating FAs as multi-phase detection failures.

According to 2HT model, an item may trigger an *old-detection state* ($\rho ^{(\textrm{old})}$) if it provides sufficient evidence of a memory match, a *new-detection state* ($\rho ^{(\textrm{new})}$) if it provides strong evidence of novelty, or – if neither threshold is reached – a *guessing state* ($\gamma$). This branching structure determines the observed hit and FA rates (Erdfelder et al., 2009). A hit occurs either when old-item detection succeeds or, if it fails, when the participant guesses *old*:

$$ \Pr (\textrm{H}) = \rho ^{(\textrm{old})} + (1 - \rho ^{(\textrm{old})})\gamma . $$

A false alarm occurs when new-item detection fails and the participant guesses *old*:

$$ \Pr (\textrm{FA}) = (1 - \rho ^{(\textrm{new})})\gamma . $$

To ensure identifiability, standard 2HT formulation assumes that the same underlying memory evidence supports old-item and new-item detection, yielding the identity $\rho ^{(\textrm{old})} = \rho ^{(\textrm{new})}$. Under designs with matched target and lure conditions, this assumption allows hits and FAs to jointly constrain a single detection parameter.

However, our design breaks this correspondence: old items yield three phase-specific hit rates, whereas new items provide a single FA rate. This 3:1 asymmetry prevents a direct mapping between hits and false alarms and violates the conditions under which $\rho ^{(\textrm{old})}$ and $\rho ^{(\textrm{new})}$ can be treated as a single parameter.

To restore identifiability while respecting the design logic and the theoretical commitments of the 2HT framework, we assume that mnemonic evidence is independent between phases and specific to each phase. Under this assumption, detecting that an item is *new* requires ruling out a match in each encoding phase, yielding the functional relation:

$$ \rho ^{(\textrm{new})} \equiv f\left( \rho ^{(\textrm{old})}_{1}, \rho ^{(\textrm{old})}_{2}, \rho ^{(\textrm{old})}_{3}\right) = \rho ^{(\textrm{old})}_1 \cdot \rho ^{(\textrm{old})}_2 \cdot \rho ^{(\textrm{old})}_3. $$

Intuitively, a participant may correctly determine that a new item does not match memories from the pre- and conditioning phases, but fail to do so for the post-conditioning phase, resulting in a detection failure, defined as the complement:

$$ 1 - \rho ^{(\textrm{new})} = 1 - \rho ^{(\textrm{old})}_1 \cdot \rho ^{(\textrm{old})}_2 \cdot \rho ^{(\textrm{old})}_3. $$

This formulation preserves identifiability and ensures that the single FA rate informs memory-based detection parameters in each phase.

#### Old-new responses used for analysis

When analyzing recognition-task data, all responses were coded dichotomously as *old* or *new*. In task formats where participants first made an old/new decision and then provided a confidence rating, we used their initial old/new choice only. In formats that collected confidence-qualified responses directly, we recoded *definitely/maybe old* as old and *definitely/maybe new* as new, consistent with prior work (Dunsmoor et al., 2015; Kalbe & Schwabe, 2022a).

This binarization is consistent with the all-or-none assumptions of the 2HT model, in which the cognitive system commits to an old or new judgment before confidence is expressed. As shown formally (see Supplementary Appendix B), incorporating high- and low-confidence responses under psychologically realistic assumptions does not change the implied old/new probabilities. Consequently, under these assumptions, when confidence levels are collapsed, the extended model reduces exactly to the standard 2HT formulation.

#### Hierarchical Bayesian estimation

We estimated 2HT parameters using a hierarchical Bayesian framework in which each participant has detection parameters for each encoding phase and CS type, as well as a guessing parameter for each semantic category. These individual-level parameters are modeled as draws from population distributions, assuming that all participants rely on the same underlying memory processes but may express them to different degrees. This hierarchical structure pools information across participants while allowing individual differences, yielding more stable estimates (Gelman & Hill, 2006; Nicenboim et al., 2025). A graphical representation of the statistical model, including prior distributions, is provided in Supplementary Fig. S1.

We linked latent detection and guessing processes to observed responses using a binomial likelihood. Old items belong to six experimental conditions, indexed by $c \in \{1,\dots ,6\}$, with $c \in \{1,2,3\}$ denoting CS– items and $c \in \{4,5,6\}$ denoting CS+ items presented pre-, during, and post-conditioning, respectively. New items belong to two semantic categories, indexed by $d \in \{1,2\}$, corresponding to CS– congruent ($d = 1$) and CS+ congruent ($d = 2$) lures. The model predicts hits ($y^{\text {(old)}}_{ic}$) out of $n^{\text {(old)}}_{ic}$ trials and FAs ($y^{\text {(new)}}_{id}$) out of $n^{\text {(new)}}_{id}$ trials, where $i$ indexes participants:

$$ y^{\text {(old)}}_{ic} \sim \textrm{Binomial}\!\left( n^{\text {(old)}}_{ic},\, \theta ^{\text {(old)}}_{ic}\right) , \qquad y^{\text {(new)}}_{id} \sim \textrm{Binomial}\!\left( n^{\text {(new)}}_{id},\, \theta ^{\text {(new)}}_{id}\right) . $$

Hit probability arises from either detection of an old item from condition $c$ ($\rho _{ic}^{\text {(old)}}$) or, if detection fails, guessing based on semantic category $d$ ($\gamma _{id}$):

$$ \theta ^{\text {(old)}}_{ic} = {\left\{ \begin{array}{ll} \rho ^{\text {(old)}}_{ic} + (1-\rho ^{\text {(old)}}_{ic})\,\gamma _{i1}, & c \in \{1,2,3\}, \\ \rho ^{\text {(old)}}_{ic} + (1-\rho ^{\text {(old)}}_{ic})\,\gamma _{i2}, & c \in \{4,5,6\}. \end{array}\right. } $$

FAs probability arises from guessing *old* after detection has failed, i.e., when participant fails to recognize that an item was never seen during the experiment:

$$ \theta ^{\text {(new)}}_{id} = (1 - \rho ^{\text {(new)}}_{id})\,\gamma _{id}. $$

Following the 2HT formulation, new-item detection arises from the joint contribution of memory for each phase (i.e., correctly identifying a new item as *new* requires detecting the absence of a memory match across all three encoding phases) expressed as the product of the phase-specific old-detection probabilities:

$$ \rho ^{\text {(new)}}_{id} = {\left\{ \begin{array}{ll} \rho _{i1}^{\text {(old)}} \rho _{i2}^{\text {(old)}} \rho _{i3}^{\text {(old)}} & \text {if } d = 1, \\ \rho _{i4}^{\text {(old)}} \rho _{i5}^{\text {(old)}} \rho _{i6}^{\text {(old)}} & \text {if } d = 2. \end{array}\right. } $$

Accordingly, weak memory in any phase increases reliance on guessing.

#### Logistic mixed-effects parameterization

To model how detection $\rho _{ic}$ and guessing $\gamma _{id}$ vary across phases and CS types for each participant, we expressed these probabilities through logistic mixed-effects models, analogous to generalized linear mixed models. Each logit-transformed probability was modeled with a linear predictor including fixed effects (experimental conditions), participant-level random effects, and study-level adjustments.

Detection of old items is modeled as:

$$ {\text {logit}}\!\left( \rho ^{\text {(old)}}_{ic}\right) = \boldsymbol{X}_c^{(\beta )}\left( \boldsymbol{\beta }_i + \boldsymbol{u}^{(\beta )}_{\text {[study]}}\right) + w_c\,\eta ^{(\rho )}, $$

where $\boldsymbol{X}_c^{(\beta )}$ is the design-matrix row encoding the six phase-by-CS-type conditions, $\boldsymbol{\beta }_i$ contains participant-specific vector of regression coefficients while $\boldsymbol{u}^{(\beta )}_{\text {[study]}}$ represents their between-study adjustments. The term $w_c$ is an indicator coding whether condition $c$ belongs to the semantic category of animals or tools, so that the coefficient $\eta ^{(\rho )}$ captures any residual category-level shift beyond the main design contrasts.

To express the coefficients in interpretable terms, we use the hypothesis matrix $\boldsymbol{H}^{(\beta )} = \textrm{inv}\!\left( \boldsymbol{X}^{(\beta )}\right)$, which defines orthogonal contrasts corresponding to the grand mean ($\beta _1$), differences between phases ($\beta _2,\beta _3$), and CS+ versus CS– differences within each phase ($\beta _4$–$\beta _6$):

$$\begin{aligned} \boldsymbol{H}^{(\beta )} = \begin{aligned} \quad \beta _1 \quad \beta _2 \quad \beta _3 \quad \beta _4 \quad \beta _5 \quad \beta _6 \\ \begin{array}{l} {phase_1^{CS--}}\\ {phase_2^{CS--}}\\ {phase_3^{CS--}} \\ {phase_1^{CS+}} \\ {phase_2^{CS+}} \\ {phase_3^{CS+}} \\ \end{array} \left[ \begin{array}{rrrrrr} 1/6 & -1 & 0 & -1 & 0 & 0 \\ 1/6 & 1 & -1 & 0 & -1 & 0 \\ 1/6 & 0 & 1 & 0 & 0 & -1 \\ 1/6 & -1 & 0 & 1 & 0 & 0 \\ 1/6 & 1 & -1 & 0 & 1 & 0 \\ 1/6 & 0 & 1 & 0 & 0 & 1 \\ \end{array}\right] \end{aligned} \end{aligned}$$

In the *logit* parameterization, participant-level weights $\boldsymbol{\beta }_i$ represent differences on a log-odds scale (excluding the intercept). When these log-odds are exponentiated, they yield odds ratios – an effect size that quantifies how much more likely an event is in one condition compared to another. For example, a log-odds of $0.18$ implies that the odds of memory detection in the CS+ condition are $e^{0.18} \approx 1.2$ times higher than in the CS– condition. Participant-level parameters $\boldsymbol{\beta }_i$ are drawn from a multivariate normal with population-level means $\boldsymbol{\mu }^{(\beta )}$ and covariance $\boldsymbol{\Sigma }^{(\beta )}$. We used weakly informative priors: Normal(0, 1) for most parameters; Normal (–0.5, 1.5) for the grand mean (based on prior predictive checks); Lewandowski–Kurowicka–Joe (shape = 2) and gamma (shape = 5, rate = 10) priors for the correlation matrix and standard deviations.

Guessing is parameterized analogously:

$$ {\text {logit}}\!\left( \gamma _{id}\right) = \boldsymbol{X}_d^{(\alpha )}\left( \boldsymbol{\alpha }_i + \boldsymbol{u}^{(\alpha )}_{\text {[study]}}\right) + w_d\,\eta ^{(\gamma )} , $$

with a hypothesis matrix:

$$ \boldsymbol{H}^{(\alpha )} = \begin{aligned} \quad \alpha _1 \quad \alpha _2 \\ \begin{array}{l} \textit{CS--} \\ \textit{CS+} \\ \end{array} \left[ \begin{array}{rrrrrr} 1/2 & -1 \\ & 1/2 & 1 \\ \end{array}\right] \end{aligned} $$

Here, $\alpha _1$ reflects overall guessing and $\alpha _2$ reflects the CS+ versus CS– contrast. Priors mirror those used for detection, except for the grand mean of guessing, which follows a Normal (–0.8, 1.5) distribution calibrated through prior predictive checks.

#### Situating the 2HT model within alternative recognition models

To contextualize the chosen recognition model, we compared the 2HT model with a one-high-threshold (1HT) variant and a signal detection theory (SDT) model using cross-validated predictive accuracy. Because the same hit and FA data constrain all three models, large differences in predictive performance are not expected (see the Supplement). Our use of 2HT is therefore motivated not by predictive superiority but by its theoretical structure and its capacity to yield identifiable, phase-specific detection processes that link hits and FAs through shared memory mechanisms. This feature allows the single FA rate to constrain the three phase-specific hit rates, which 1HT cannot do because FAs contain no memory signal, and SDT cannot do cleanly because its single decision criterion couples discriminability and bias across phases. For these reasons, 2HT provides the most appropriate foundation for our analyses.

#### Visualization and exploratory clustering of memory-based detection estimates

To visualize the high-dimensional structure of individual heterogeneity in memory-based detection, we applied dimensionality reduction and clustering to participants’ posterior detection estimates. Clustering was performed on the maximum a posteriori (MAP) estimates of the participant-level detection weights (i.e., $\boldsymbol{\beta _i}$), providing a representative six-dimensional vector. This analysis was descriptive rather than inferential, aiming to visualize variation in detection profiles and explore potential subgroup structure.

We used Kohonen self-organizing maps (SOMs) via the kohonen R package (Kohonen, 1982; Wehrens & Kruisselbrink, 2018) to project multivariate data onto a two-dimensional grid while preserving topological structure (Doshi & Konkle, 2023; Graziano & Aflalo, 2007). SOMs provided a straightforward way to convert complex, multi-dimensional parameter estimates into an interpretable two-dimensional layout. First, they place similar participants close together on the map, making broad patterns or potential groupings easier to identify. Second, SOMs yield a stable, grid-like representation that is easier to interpret than the results of visualization tools, which vary across runs. Finally, because SOMs do not require additional modeling assumptions, they offer a simple descriptive means of examining whether the data contain any naturally occurring subgroups.

We trained a 7 x 8 hexagonal SOM (no edge wrapping) for 5000 iterations on scaled MAP estimates. All nodes were populated, indicating good coverage. We then performed exploratory clustering by applying k-means to a Euclidean distance matrix of node vectors. The silhouette method indicated two clusters, which was confirmed by clustering the original $\boldsymbol{\beta }_i$ vectors. These results are exploratory; formal inference is provided by the latent mixture model below.

#### Modeling latent individual heterogeneity in memory-based detection

Exploratory analyses suggested that a single population distribution may not capture detection parameters. We therefore extended the hierarchical 2HT model with a latent mixture component. A latent mixture model assumes that data arise from multiple unobserved subpopulations with distinct parameter distributions (Mcelree, 2000; Nicenboim & Vasishth, 2018; Ollman, 1966). In the present context, each latent component corresponds to a distinct pattern of phase- and CS-specific detection parameters in the 2HT model – patterns we refer to as memory profiles. These profiles are not psychological constructs on their own; rather, they summarize structured variation in the 2HT detection parameters across individuals, indicating a distinct organization of episodic memories for emotional and nearby neutral events.

Critically, this approach enables inference directly on latent memory processes rather than observed responses, which conflate mnemonic with non-mnemonic tendencies. As a result, we can evaluate whether individuals differ not merely in overall recognition performance but in the mnemonic component of recognition decisions. Moreover, unlike clustering methods that impose an arbitrary number of subgroups, mixture modeling tests the theoretically relevant question of whether memory-based detection is better described by distinct latent subgroups or by a single homogeneous group. This aligns the statistical procedure with the inferential goal – determining whether heterogeneity in emotional-memory effects reflects distinct modes of cognitive processing. A graphical representation of the mixture model, including the data-generating structure and all prior distributions, is provided in Supplementary Fig. S1.

We extended the baseline model with a mixture following established approaches in cognitive modeling (Bartlema et al., 2014; Lee & Stark, 2023). Each participant has a latent membership variable ($z_i$), which assigns them probabilistically to one of two latent components:

$$ \begin{aligned} z_i&\sim \text {Bernoulli}(\phi ), \end{aligned} $$

with a uniform Beta prior on the mixing proportion $\phi$, reflecting no prior preference for either subgroup. Conditional on group membership, each participant’s detection-weight vector $\boldsymbol{\beta }_i$ is drawn from one of two multivariate normal distributions with distinct component-specific means $\boldsymbol{\mu }^{(\beta , z_i)}$ but a shared diagonal covariance matrix $\Sigma ^{(\beta )}$ assuming common variance:

$$ \begin{aligned} \boldsymbol{\beta }_{i} \mid z_i&\sim \text {MvNormal}(\boldsymbol{\mu }^{(\beta ,\ z_i)}, \Sigma ^{(\beta )}). \end{aligned} $$

This formulation preserves the structure of the baseline hierarchical model while allowing for systematic differences in memory-based detection across latent subgroups. Importantly, participants are not deterministically classified; the posterior distribution over $z_i$ quantifies the uncertainty inherent in assigning individuals to the latent subpopulations.

Model adequacy was evaluated via three complementary procedures: (i) cross-validated predictive assessment comparing the mixture model with the baseline model, (ii) study-wise refits to assess parameter replicability across independent datasets, and (iii) the consistency of posterior group membership across studies (for procedural details, see the Supplement). To aid interpretation of the latent subgroups, we examined associations between posterior group membership and individual differences in Pavlovian learning, including Rescorla-Wagner learning rate and anticipatory arousal (see the Supplement).

**Fig. 2:**
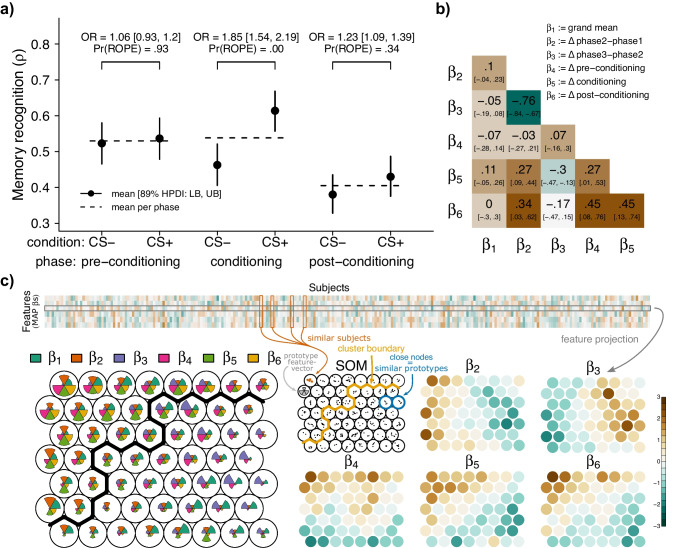
Bayesian two-high-threshold (2HT) memory-based detection estimates and their structure revealed through correlations and exploratory clustering. **a**. Posterior estimates of memory-based recognition ($\rho$) across experimental conditions. *Error bars* represent 89% highest posterior density intervals (HPDIs). Condition comparisons are expressed using odds ratios (ORs), with the proportion of the posterior within the region of practical equivalence (ROPE) denoted as Pr(ROPE). **b**. Posterior correlation matrix of individual-level parameter estimates ($\boldsymbol{\beta }$), i.e., capturing effects of experimental conditions on recognition memory. *Warmer colors* indicate stronger positive correlations; *cooler colors* indicate stronger negative correlations. *Matrix values* represent mean posterior correlations with associated 89% HPDIs. **c**. Individual differences in memory recognition projected onto a two-dimensional grid using Kohonen self-organizing maps (SOMs). Each node represents a prototype capturing similar observations; spatial proximity reflects similarity in the underlying feature space. The *black border* indicates two participant clusters identified via k-means. Accompanying individual feature maps show the distribution of each (scaled) feature across the SOM grid, illustrating multivariate relationships among parameters

#### Bayesian inference

All Bayesian analyses were conducted in Stan (Stan Development, 2023) via the rstan R package (Stan Development Teamt, 2024), using the No-U-Turn Sampler (Betancourt, 2018; Hoffman & Gelman, 2014). For each model, four MCMC chains were run for 20,000 iterations each (2000 warm-up, thinning = 2), yielding 40,000 post-warm-up samples. Convergence was evaluated via traceplots, $\hat{R}$ statistics (Brooks & Gelman, 1998), and effective sample sizes (>10,000 for all key parameters).

Model adequacy was assessed through posterior predictive checks comparing simulated and observed hit and FA rates, both overall and separately by study. For the mixture model, checks were additionally conditioned on latent group membership to evaluate whether both subgroups were supported by the data. Generalizability of the inferred group structure across studies was examined by comparing study-specific posterior predictive distributions (see the Supplement).

We report posterior means and 89% highest posterior density intervals (HPDIs) in the format: mean [lower bound, upper bound]. Evidence for or against effects was quantified using the Region of Practical Equivalence (ROPE) (Etz et al., 2024; Kruschke, 2018; Makowski et al., 2019), with standard ranges applied to the log-odds (odds ratio): [−0.18, 0.18] ($\approx$ [0.85, 1.20]) and correlations: [-0.1, 0.1] (Cohen, 2013; Kruschke, 2014). For the mixing proportion, we specified a ROPE of [0, 0.2], where values near zero indicate no meaningful subgroup.

**Table 1:** Results of the two-high-threshold (2HT) model with latent mixture extension

Subgroup	Phase	CS–	CS+	Odds ratio	Pr(ROPE)
1	pre-conditioning	.48 [.42, .55]	.51 [.45, .57]	1.11 [0.96, 1.29]	.81
	conditioning	.54 [.48, .61]	.74 [.68, .79]	2.36 [1.86, 2.99]	.00
	post-conditioning	.33 [.28, .39]	.41 [.35, .47]	1.42 [1.21, 1.69]	.05
2	pre-conditioning	.55 [.49, .61]	.55 [.49, .61]	1.01 [0.88, 1.16]	.94
	conditioning	.40 [.34, .46]	.50 [.43, .56]	1.51 [1.20, 1.87]	.05
	post-conditioning	.42 [.36, .48]	.44 [.38, .50]	1.10 [0.95, 1.28]	.81

*Note.* Values in CS+ and CS– columns indicate recognition probability estimates. *Square brackets* indicate the 89% highest posterior density interval (HPDI), representing the most credible range of parameter estimates

We compared the baseline and mixture models using five-fold cross-validation stratified by participant and study. Models were trained on 80% of participants and evaluated on the remaining 20%, with each participant held out once. Predictive performance was quantified using the expected log predictive density (ELPD) computed via the loo R package (Vehtari et al., 2024). Model comparisons were based on differences in ELPD; differences exceeding twice their standard error indicated superior predictive accuracy (Vehtari et al., 2016).

## Results

### Selective memory enhancement by emotional learning varies across phases and individuals

The baseline 2HT model yielded results largely consistent with the original publication (Kalbe & Schwabe 2022a) as illustrated in Fig. 2a. The individual observed hits and FAs underlying the model are shown in Supplementary Fig. S5. Memory-based detection probability was higher for CS+ than CS– items encoded during conditioning ($\rho _{\text {CS}^+}$ = .61 [.56, .67]; $\rho _{\text {CS}^-}$ = .46 [.41, .52]), revealing robust category-selective memory prioritization (OR = 1.85 [1.54, 2.19], Pr(ROPE) = .00), which did not generalize clearly to other phases. Detection rates for pre-conditioning CS+ and CS– items were nearly identical ($\rho _{\text {CS}^+}$ = .54 [.48, .59]; $\rho _{\text {CS}^-}$ = .52 [.47, .58]; OR = 1.06 [0.93, 1.2], Pr(ROPE) = .93). Post-conditioning CS+ items showed slight advantage ($\rho _{\text {CS}^+}$ = .43 [.38, .49]; $\rho _{\text {CS}^-}$ = .38 [.33, .44]), but overlapped with the ROPE (OR = 1.23 [1.09, 1.39], Pr(ROPE) = .34), providing tentative evidence for proactive effects. These results were corroborated by both the 1HT and SDT models (see the Supplement).

Participant-level correlations revealed individual differences in category-selective memory prioritization (Fig. 2b). This was most evident between conditioning and post-conditioning phases (*r* = .45 [.13, .74], Pr(ROPE) = .04), with a tentative effect of smaller magnitude between conditioning and pre-conditioning (*r* = .27 [.01, .53], Pr(ROPE) = .14). In other words, individuals who selectively prioritized memory for items encoded during conditioning were more likely to do so post-conditioning, and to a lesser extent, pre-conditioning. We also observed a strong negative correlation between phases regardless of CS type (*r* = -0.76 [-0.84, -0.67], Pr(ROPE) = .00), highlighting an additional source of individual variability and motivating further exploration through clustering with Kohonen self-organizing maps. The analysis revealed a two-cluster structure (Fig. 2c), which we examined using individual feature maps, each reflecting similarity among participants through spatial proximity. Specifically, maps of the grand average, phase effects, and CS+ vs. CS– differences uncovered three axes of variation: (a) a northwest-southeast gradient of category-selective prioritization (particularly during and post-conditioning); (b) a west-east gradient representing overall phase effects; and (c) a diffuse pattern of the grand average suggesting its independence.

Together, the results showed that emotional learning selectively prioritized episodic memory for items encoded during conditioning, alongside individual differences – particularly in selective prioritization and phase-related effects – that clustered into two distinct subgroups.

### Emotional learning leads to two latent episodic memory profiles

**Fig. 3:**
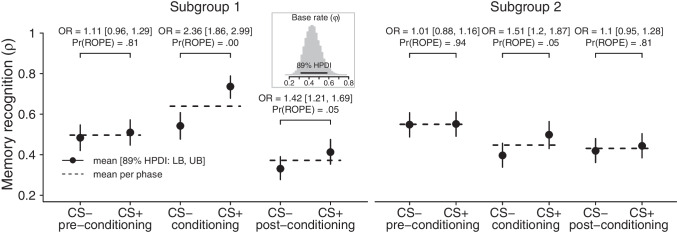
Bayesian two-high-threshold (2HT) mixture model infers two latent memory-based detection profiles. Posterior estimates of memory-based recognition ($\rho$) across experimental conditions per subgroup. *Error bars* represent 89% highest posterior density intervals (HPDIs). Condition comparisons are expressed using odds ratios (ORs), with the proportion of the posterior within the region of practical equivalence (ROPE) denoted as Pr(ROPE). The inset plot depicts posterior proportion of the subgroups

We formally tested for latent structure in memory-based recognition by extending the 2HT model to probabilistically assign participants to two subgroups, as indicated by the clustering. The extended model outperformed the non-mixture model on held-out data ($\Delta \text {ELPD}$ = 202.94, SE = 33.65, *z* = 6.03). The estimated subgroup proportion was 0.44 [0.32, 0.58], consistent with the two-cluster solution (Pr(ROPE) = .00). These subgroups were present across all four constituent studies, suggesting they were not driven by minor variations in experimental procedures (see the Supplement). Furthermore, group membership was unrelated to individual differences in Rescorla-Wagner learning rate (*r* = 0 [-0.04, 0.04], Pr(ROPE) = 1) or differential anticipatory SCRs (*r* = -0.05 [-0.09, -0.01], Pr(ROPE) = 0.97), despite the learning rate accounting for variation in category-selective memory prioritization for items encoded during conditioning within the baseline 2HT model (see the Supplement). Both subgroups exhibited similar Pavlovian learning, with overlapping distributions of learning rates (five-number summary: Subgroup 1 = [0.01, 0.13, 0.19, 0.24, 0.53]; Subgroup 2 = [0.01, 0.12, 0.2, 0.26, 0.44]) and differential anticipatory SCRs (Subgroup 1 = [-0.26, 0, 0.02, 0.1, 0.71]; Subgroup 2 = [-0.11, 0, 0.04, 0.12, 0.78]). Overall memory recognition was comparable between subgroups (OR = 1.13 [1.01, 1.3], Pr(ROPE) = .8), yet a breakdown by condition revealed distinct memory profiles (Table 1 and Fig. 3). These results suggest that subgroups differed in how emotional learning shaped episodic memory, rather than in associative learning performance or overall mnemonic capacity.

#### Profile 1: Memory structured around emotional relevance

Subgroup 1 showed strong category-selective memory prioritization for items encoded during conditioning (OR = 2.36 [1.86, 2.99], Pr(ROPE) = .00), which generalized to the items encoded afterward (OR = 1.42 [1.21, 1.69], Pr(ROPE) = .05), but not beforehand (OR = 1.11 [0.96, 1.29], Pr(ROPE) = .81). When collapsing across stimulus type, recognition was highest for conditioning-phase items compared to pre- and post-conditioning phases (conditioning vs. pre-conditioning: OR = 3.39 [2.22, 5.17], Pr(ROPE) = .00; conditioning vs. post-conditioning: OR = 9.09 [6.67, 14.29], Pr(ROPE) = .00). A similar pattern was observed for CS– items alone, ruling out the possibility that the phase effect was driven solely by increased recognition of CS+ items (conditioning vs. pre-conditioning: OR = 1.27 [0.99, 1.61], Pr(ROPE) = .36; conditioning vs. post-conditioning: OR = 2.39 [1.9, 3.02], Pr(ROPE) = .00).

#### Profile 2: Memory structured around nominal temporal boundaries

Subgroup 2 showed an attenuated category-selective enhancement for the items encoded during conditioning (OR = 1.51 [1.2, 1.87], Pr(ROPE) = .05), without generalization to pre- (OR = 1.01 [0.88, 1.16], Pr(ROPE) = .94), or post-conditioning (OR = 1.1 [0.95, 1.28], Pr(ROPE) = .81). This subgroup also displayed the effect of phase but in reverse: recognition was highest for items encoded before conditioning, then declined across conditioning and post-conditioning (pre-conditioning vs. conditioning: OR = 2.27 [1.67, 3], Pr(ROPE) = .00; conditioning vs. post-conditioning: OR = 1.14 [0.78, 2], Pr(ROPE) = .48). Again, CS– items resembled the pattern, suggesting a general phase-related decrease (pre-conditioning vs. conditioning: OR = 1.85 [1.52, 2.26], Pr(ROPE) = .00; conditioning vs. post-conditioning: OR = 0.91 [0.73, 1.11], Pr(ROPE) = .74).

Together, two memory profiles emerged: one organized primarily around behavioral relevance, with prioritization extending beyond nominal event boundaries, and another organized primarily around temporal structure, with enhancement confined to the conditioning episode.

## Discussion

Emotional experiences are often well retained because they support adaptive behavior (Nairne et al., 2007; Shohamy & Adcock, 2010). Yet emotions may do more than strengthen memory; they may reorganize it (Horwath et al., 2023). Episodic representations are shaped by both temporal segmentation and motivational relevance – two forces that can diverge when emotionally significant information emerges within ongoing experience. Does emotional learning remain confined to its temporal context, or does it extend across nominal event boundaries to alter memory for adjacent neutral events? To address this question, we quantified individual variability in how aversive learning prioritized semantically related neutral information encoded before and after threat conditioning. Although all participants demonstrated comparable Pavlovian learning, their memory patterns revealed two latent profiles. These profiles were consistent with two organizational modes: one preserving boundary-based segmentation, and another favoring relevance-weighted continuity across time.

In one subgroup, memory performance closely tracked nominal temporal context. Participants showed a primacy-like advantage for the first encoding phase, with better memory for pre-conditioning items than for conditioning items despite the latter’s emotional salience. Emotional learning enhanced memory selectively within the conditioning phase itself but did not generalize across phases, consistent with the presence of brief temporal gaps. This pattern aligns with event segmentation accounts, in which perceptual boundaries constrain memory integration and guard against overgeneralization (Dunsmoor et al., 2018). For these individuals, episodic organization appears to prioritize temporal information, with emotional salience modulating memory strength locally without reshaping the broader boundary-defined organization of experience.

In contrast, a second subgroup exhibited a pattern consistent with relevance-weighted contextual organization. In these individuals, emotional learning enhanced memory not only for items encoded during conditioning but also selectively for semantically related items encoded after conditioning, despite the intervening temporal boundary. This forward mnemonic generalization suggests that emotional salience increases continuity between the emotional episode and the subsequent neutral experience. Such a “spillover” effect aligns with models proposing that emotional states persist over time and promote memory integration (Clewett & McClay, 2024; Mcclay & Sachs, 2023; Tambini et al., 2016). For these individuals, emotional relevance appears to partially override event boundaries, reorganizing memory according to motivational significance rather than temporal proximity alone.

This cross-boundary influence was asymmetric, as re-analysis using cognitive modeling that accounted for individual variability revealed no evidence of retroactive enhancement, consistent with the null group-level findings reported by Kalbe and Schwabe (2022a). One possible explanation is that, in our dataset, pre-conditioning items were already well remembered in both groups, leaving limited opportunity for mechanisms supporting retroactive enhancement, such as behavioral tagging – a process proposed to preferentially stabilize weak memories if they occur near a salient event (Cowan et al., 2021; Dunsmoor et al., 2022). This interpretation aligns with evidence suggesting that retroactive enhancement depends on the availability of relatively unstable memories that would otherwise be forgotten (Ballarini et al., 2009; Frey & Morris, 1997). Notably, Dunsmoor et al. (Dunsmoor et al., 2015) reported retroactive effects under standard encoding conditions but found that these effects were eliminated when pre-conditioning items were strengthened through repetition. Future work may therefore benefit from ensuring relatively weak encoding of pre-conditioning material when examining retroactive effects attributable to behavioral tagging – for example, by reducing stimulus presentation times or increasing the number of stimuli presented.

The memory profiles were not predicted by observable indices of emotional learning (skin conductance responses and Rescorla-Wagner learning rate) or by overall recognition performance. Importantly, this absence of behavioral differences does not render the profiles uninterpretable. On the contrary, because both groups performed comparably on these standard behavioral metrics under identical external conditions, the latent structure cannot be reduced to general performance disparities, arousal differences, or differential task engagement. Instead, these findings suggest that subgroup differences reflect variability in how emotional salience shapes contextual dynamics rather than how strongly Pavlovian associations are acquired.

Within a framework of emotional context maintenance and retrieval models (Talmi et al., 2019), such variability may arise from differences in the persistence of emotion-modulated context representations, including the extent to which affective states carry over beyond the emotional episode, alter perceived event boundaries, or bias post-encoding consolidation processes (Lilja et al., 2024; Mcclay & Sachs, 2023). Related work demonstrates that emotional learning can distort source memory, increasing misattributions of semantically related items encoded before and after conditioning to the learning episode itself (Laing et al., 2026). These distortions may coincide with greater contextual blending, which may promote cross-boundary mnemonic generalization of emotional learning. In certain contexts, such generalization may be adaptive, supporting the capacity to infer danger from memories beyond direct aversive experience (Baczkowski et al., 2023).

### Limitations

We inferred latent episodic organization from memory recognition of items encoded across experimentally defined phases rather than from direct measures of subjective event segmentation. The boundaries separating phases were nominal, and we did not assess whether participants experienced them as meaningful transitions, nor did we collect source memory measures that would allow us to examine contextual binding directly. Accordingly, the identified profiles are derived from internal model structure rather than external measures (e.g., affective, personality, or cognitive individual differences). As a result, our conclusions concern how emotional learning shaped memory across experimentally segmented phases, and do not allow us to determine how latent memory profiles map onto broader cognitive or affective characteristics. Future work incorporating direct measures of both contextual processing and individual differences will be important for clarifying the mechanisms and functional significance of these profiles.

### Conclusion

The present findings suggest that episodic memory reflects an interaction between externally imposed event boundaries and internally evolving state tracking emotional relevance. Critically, cross-boundary mnemonic generalization emerged when the emotional episode was strongly prioritized in memory. In these individuals, emotional learning enhanced memory not only within the conditioning phase but also for semantically related information encoded afterward, despite an intervening temporal boundary – consistent with the idea that emotional salience can prolong an affectively biased contextual state. When the emotional episode was assigned a lower mnemonic priority, emotional memory enhancement remained confined to the emotional context, and memory performance tracked nominal temporal structure. Emotional learning therefore neither uniformly overrides event boundaries nor is uniformly constrained by them. Instead, its cross-boundary impact appears conditional on how prominently the emotional episode is represented in memory.

## Supplementary Information

Below is the link to the electronic supplementary material.

Supplementary file 1 (pdf 388 KB)

## Data Availability

This article is based on the primary data published in Kalbe & Schwabe (2022a) and publicly available at https://osf.io/qpm3t. Demographic data and secondary data, including skin conductance response, are available at https://doi.org/10.5281/zenodo.18661964. The study materials are not publicly available due to licensing restrictions.

## Code Availability

Custom analysis scripts and their outputs (e.g., posterior MCMC samples and diagnostics) are available at https://doi.org/10.5281/zenodo.18661964.

## Declarations

### General

Artificial intelligence: ChatGPT (OpenAI, Inc., US) was used for language editing to improve clarity, flow, and conciseness. The authors take full responsibility for the content of the published article.

### Preregistration

The fourth study included in the original report was preregistered prior to data collection at https://osf.io/9hzmk. The current analysis plan was not preregistered.
