# Supplementary material for: Divergent modes of episodic organization underlie whether emotional learning enhances memory across event boundaries
Source: Psychon Bull Rev. 2026 Jun 8;33(5):170. doi: 10.3758/s13423-026-02924-5 (PMC13246875; doi:10.3758/s13423-026-02924-5)
Supplement: Supplementary file 1 — (pdf 388 KB) [file 13423_2026_2924_MOESM1_ESM.pdf]

Supplementary Material for: *Divergent modes of episodic organization underlie whether emotional learning enhances memory across event boundaries*

Blazej M. Baczkowski[1], Michiko Sakaki[1,2], Felix Kalbe[3], Kou Murayama[1,2], and Lars Schwabe[3]
[1]Hector Research Institute of Education Sciences and Psychology, University of Tübingen
[2]Research Institute, Kochi University of Technology
[3]Department of Cognitive Psychology, University of Hamburg

Supplementary Methods

Latent-mixture two-high-threshold model (2HT) checks

To evaluate the latent-mixture 2HT model, we conducted two complementary analyses that operate at different levels. First, we assessed parameter-level replicability across independent datasets by fitting the mixture model separately to each study and comparing the resulting posterior distributions. This tests whether the component-defining effects are reproducible when the model is refit in isolation. Second, using the full pooled dataset, we examined the generalizability of the latent class structure itself by studying the distribution of posterior group membership probabilities across studies. This tests whether both latent subpopulations are represented within each study when the model is estimated globally, i.e., across the entire dataset. Together, these analyses provide convergent evidence about the stability and generalizability of the mixture solution.

Parameter replicability across independent datasets

To determine whether the latent subgroup structure reflects reproducible effects rather than study-specific noise, we fit the latent-mixture 2HT model separately to each of the three larger studies in the dataset (i.e., study 2, study 3, and study 4). We then quantified the similarity of the resulting posterior parameter estimates using a density-overlap metric:

$$\kappa(p_1, p_2) = \int_{-\infty}^{\infty} \min\{p_1(x),\ p_2(x)\}\, dx.$$

Posterior densities were computed on a shared grid and overlap values were averaged across all pairwise study comparisons. This analysis evaluates whether the effects defining the latent components are consistent when the model is re-estimated independently for each dataset.

Posterior group membership in the model with full dataset

To complement the parameter-replicability analysis, we next examined the generalizability of the latent class solution when the model is fit to the full dataset. Even if parameter estimates are consistent across independent studies, a mixture model may still assign participants unevenly across studies when estimated globally. We therefore evaluated whether both latent groups were represented within each study and whether their posterior membership distributions were comparable.

Correspondence concerning this article should be addressed to Blazej M. Baczkowski, blazej.baczkowski@uni-tuebingen.de, University of Tübingen.

Figure S1

Graphical model representation of the Bayesian two-high-threshold model for old–new recognition judgments including latent mixture extension.

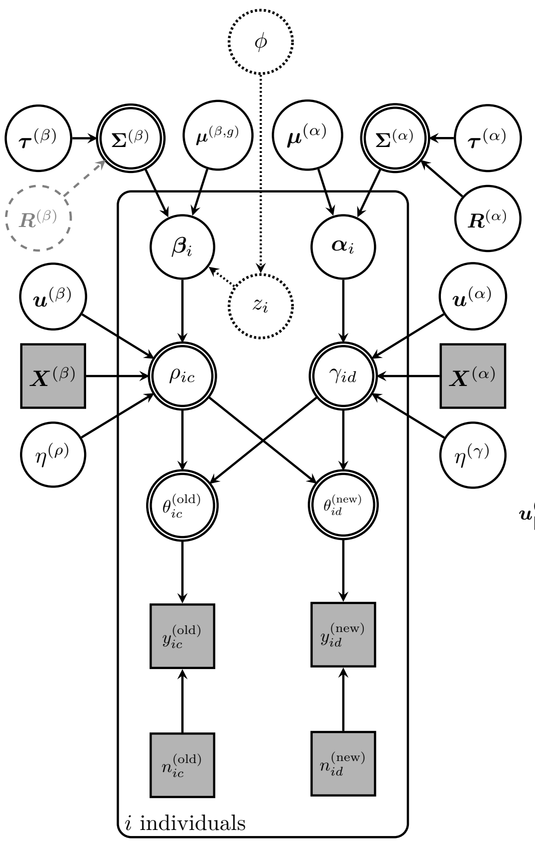

$$
\begin{aligned}
\phi &\sim \text{Beta}(1,1) \quad \text{(mixture)} \\
z_i &\sim \text{Bernoulli}(\phi)^*,\ z_i \in \{0,1\} \quad \text{(mixture)} \\
\mu_1^{(\beta,g)} &\sim \begin{cases} \text{Normal}(-0.5, 1.5),\ g = 1 & \text{(baseline)} \\ \text{Normal}(-0.5, 1.5),\ g \in \{0,1\} & \text{(mixture)} \end{cases} \\
\mu_j^{(\beta,g)} &\sim \begin{cases} \text{Normal}(0,1),\ j \in \{2{:}6\},\ g = 1 & \text{(baseline)} \\ \text{Normal}(0,1),\ j \in \{2{:}6\},\ g \in \{0,1\} & \text{(mixture)} \end{cases} \\
\mu_1^{(\alpha)} &\sim \text{Normal}(-0.8, 1.5) \\
\mu_2^{(\alpha)} &\sim \text{Normal}(0,1) \\
\tau_j^{(\beta)} &\sim \text{Gamma}(5,10),\ j \in \{1{:}6\} \\
\tau_k^{(\alpha)} &\sim \text{Gamma}(5,10),\ k \in \{1,2\} \\
\boldsymbol{R}^{(\beta)} &\sim \text{LKJcorr}(2),\ \boldsymbol{R}^{(\beta)} \in \mathbb{R}^{6\times 6} \\
\boldsymbol{R}^{(\alpha)} &\sim \text{LKJcorr}(2),\ \boldsymbol{R}^{(\alpha)} \in \mathbb{R}^{2\times 2} \\
\boldsymbol{\Sigma}^{(\beta)} &= \begin{cases} \text{diag}(\boldsymbol{\tau}^{(\beta)}) \cdot \boldsymbol{R}^{(\beta)} \cdot \text{diag}(\boldsymbol{\tau}^{(\beta)}) & \text{(baseline)} \\ \text{diag}(\boldsymbol{\tau}^{(\beta)})^2 & \text{(mixture)} \end{cases} \\
\boldsymbol{\Sigma}^{(\alpha)} &= \text{diag}(\boldsymbol{\tau}^{(\alpha)}) \cdot \boldsymbol{R}^{(\alpha)} \cdot \text{diag}(\boldsymbol{\tau}^{(\alpha)}) \\
\boldsymbol{\beta}_i \mid z_i &\sim \text{MvNormal}(\boldsymbol{\mu}^{(\beta,z_i)},\ \boldsymbol{\Sigma}^{(\beta)}),\ \boldsymbol{\beta}_i \in \mathbb{R}^{6\times 1} \\
\boldsymbol{\alpha}_i &\sim \text{MvNormal}(\boldsymbol{\mu}^{(\alpha)},\ \boldsymbol{\Sigma}^{(\alpha)}),\ \boldsymbol{\alpha}_i \in \mathbb{R}^{2\times 1} \\
\omega_j^{(\beta)},\ \omega_k^{(\alpha)} &\sim \text{Uniform}(0.01, 0.4),\ j \in \{1{:}6\},\ k \in \{1,2\} \\
u_{j[study]}^{(\beta)} &\sim \text{Normal}(0, \omega_j^{(\beta)}),\ study \in \{1{:}4\} \\
u_{k[study]}^{(\alpha)} &\sim \text{Normal}(0, \omega_k^{(\alpha)}),\ study \in \{1{:}4\} \\
\boldsymbol{u}_{[study]}^{(\beta)},\ \boldsymbol{u}_{[study]}^{(\alpha)} &= [u_{1[study]}^{(\beta)}, \ldots, u_{6[study]}^{(\beta)}]^\top,\ [u_{1[study]}^{(\alpha)}, u_{2[study]}^{(\alpha)}]^\top \\
c &= \begin{cases} 1,2,3 & \text{for phases 1–3}^{(\text{CS}-)} \\ 4,5,6 & \text{for phases 1–3}^{(\text{CS}+)} \end{cases} \\
d &= 1 \text{ for CS}-,\ 2 \text{ for CS}+ \\
w_c,\ w_d &= \begin{cases} -1/2 & \text{if } c,d \in \{\text{animals}\} \\ 1/2 & \text{if } c,d \in \{\text{tools}\} \end{cases} \\
\eta^{(\rho)},\ \eta^{(\gamma)} &\sim \text{Normal}(0,1) \\
\text{logit}(\rho_{ic}) &= \boldsymbol{X}_c^{(\beta)} \cdot (\boldsymbol{\beta}_i + \boldsymbol{u}_{[study]}^{(\beta)}) + w_c\ \eta^{(\rho)},\ \boldsymbol{X}_c^{(\beta)} \in \mathbb{R}^{1\times 6} \\
\text{logit}(\gamma_{id}) &= \boldsymbol{X}_d^{(\alpha)} \cdot (\boldsymbol{\alpha}_i + \boldsymbol{u}_{[study]}^{(\alpha)}) + w_d\ \eta^{(\gamma)},\ \boldsymbol{X}_d^{(\alpha)} \in \mathbb{R}^{1\times 2} \\
\theta_{ic}^{(\text{old})} &= \begin{cases} \rho_{ic} + (1-\rho_{ic})\ \gamma_{i1}, & c \in \{1,2,3\} \\ \rho_{ic} + (1-\rho_{ic})\ \gamma_{i2}, & c \in \{4,5,6\} \end{cases} \\
\theta_{id}^{(\text{new})} &= \begin{cases} (1-\rho_{i1}\ \rho_{i2}\ \rho_{i3})\ \gamma_{i1} & \text{if } d = 1 \\ (1-\rho_{i4}\ \rho_{i5}\ \rho_{i6})\ \gamma_{i2} & \text{if } d = 2 \end{cases} \\
y_{ic}^{(\text{old})} &\sim \text{Binomial}(\theta_{ic}^{(\text{old})}, n_{ic}^{(\text{old})}),\ c \in \{1{:}6\} \\
y_{id}^{(\text{new})} &\sim \text{Binomial}(\theta_{id}^{(\text{new})}, n_{id}^{(\text{new})}),\ d \in \{1,2\}
\end{aligned}
$$

Note. The left panel depicts a graphical model to illustrate the dependencies between observed data and latent parameters with nodes and directed edges. Nodes represent random variables, which can be either observed (shaded) or unobserved (unshaded), and either continuous (round) or discrete (square). Edges denote probabilistic or deterministic relationships. Solid nodes and edges are shared across both baseline and latent mixture models. The dashed grey node is present only in the baseline model, while dotted nodes appear exclusively in the mixture extension. Participant-level information is enclosed within a plate, indicating that the graphical structure is replicated across participants. The right panel details the generative model, specifying the assumed probability distributions over random variables and the deterministic equations that define their dependencies. The parameter ϕ represents the base rate of each qualitatively distinct latent participant group, and z indicates a discrete group assignment variable, shown here for clarity. In practice, z was marginalized out during model estimation.

We extracted posterior group membership probabilities for each participant and summarized both hard class assignments and soft probabilities by study. This helped identify studies where one group may have been underrepresented or absent, potentially indicating sampling bias or limited within-study variability.

First, we calculated the proportion of participants assigned to each group (via hard assignments) per study to check for trivial or skewed distributions. Next, we plotted empirical cumulative distribution functions (ECDFs) of the average posterior probabilities to examine the separation between groups, expecting bimodal patterns near 0 and 1. Finally, we used the Kolmogorov–Smirnov (KS) statistic to compare the similarity of posterior probability distributions across studies. This statistic, defined as the maximum distance between two ECDFs, serves as a descriptive measure of the difference between distributions, ranging from 0 (identical) to 1 (completely distinct).

At each MCMC draw s we computed the KS distance between the posterior group membership probabilities for participants in two studies:

$$KS^{(s)} = D(p^{(s)}_{z[S_1]}, p^{(s)}_{z[S_2]})$$

where D denotes the distance between the two samples, and $p^{(s)}_{z[S]}$ is the vector of posterior probabilities of group membership for participants in particular study S at draw s. Because the KS statistic is sensitive to sample size, larger studies were subsampled to match the smaller ones at each draw, yielding a posterior distribution over KS values. This analysis evaluates whether the latent groups inferred from the pooled model are present and well separated across all studies.

Latent-mixture two-high-threshold model (2HT): Posterior group membership and association with participant-level covariates

We explored whether latent subgroup membership was associated with individual differences in learning rate as described by Rescorla-Wagner rule and anticipatory skin conductance response during conditioning.

Learning rate

We used trial-wise binary shock expectancy ratings during conditioning to estimate individual learning rates, based on a Rescorla-Wagner (RW) learning model (Rescorla & Wagner, 1972; Tzovara et al., 2018):

$$x_t = x_{t-1} + \alpha(u_{t-1} - x_{t-1}).$$

Here, the associative strength x_t represents the predicted likelihood of an outcome and is updated based on the prediction error – the difference between the previous prediction x_{t-1} and the actual outcome u_{t-1}, where u_t is binary (1 = shock, 0 = no shock). The prediction error is scaled by a subject-specific learning rate α $(0 < \alpha < 1)$, which determines the rate of update based on outcomes of previous trials. In our setup, higher learning rates correspond to faster identification of the shock-predictive category, whereas lower learning rates correspond to greater uncertainty about the category-shock contingency.

We assumed that participants had no prior expectations about the CS+ and CS−, setting the initial associative strength to 0.5 for each condition, with a common learning rate. We interpreted the associative strength x_t as the participant's belief about the probability of the US occurring, which directly mapped onto the probability of a binary response indicating shock expectation, such that $\theta_t = x_t$. Hence, the behavioural binary response *shock* vs. *no-shock* was modelled as Bernoulli-distributed outcome:

$$y_t \sim \text{Bernoulli}(\theta_t).$$

We implemented the RW rule within a hierarchical framework, in which each participant had an individual learning rate α_i, with $\text{logit}(\alpha_i)$ assumed to follow a normal distribution with population

expectation μ and variance τ^2:

$$\text{logit}(\alpha_i) \sim \text{Normal}(\mu, \tau).$$

We placed weakly informative priors on the population parameters:

$$\mu \sim \text{Normal}(0, 2),$$
$$\tau \sim \text{Gamma}(5, 10).$$

To obtain point estimates of the model parameters, we found the maximizing posterior density using the L-BFGS algorithm, implemented in the Stan language (Stan Development Team, 2023) and interfaced with the R package `rstan` (Stan Development Team, 2024).

To check for model mis-specification, we performed posterior predictive checks using graphical overlays and discrepancy measures based on simulated vs. observed summary statistics. Specifically, we computed the average proportion of shock expectancy across participants, aggregated within three trial bins per condition: trials 1–10, 11–20, and 21–30. Overall, the model tended to slightly underpredict the proportion of expected shocks compared to the observed data. However, these discrepancies were modest and fell within a reasonable range. The largest differences between predicted and observed means were approximately 0.67 vs. 0.72 for the CS+ condition and 0.03 vs. 0.12 for the CS– condition.

Visual inspection of trial-wise changes in associative strength (i.e., the model-derived probability of shock expectation) suggested that this underfitting may have stem from the use of a shared learning rate across conditions and the assumption of one-to-one correspondence between $\theta_t = x_t$. This constraint likely resulted in smoother predicted trajectories that failed to fully capture the more variable and less predictable patterns of behavioral responses observed in the empirical data.

Skin conductance response

The current study re-used condition-wise average anticipatory skin conductance responses (SCRs) during conditioning, as reported in the original study and provided by the corresponding author. Full details of the signal preprocessing and estimation procedures are described in the original report (Kalbe & Schwabe, 2022).

Analysis

To explore whether posterior group membership is associated with participant-level covariates, such as α learning rate of RW model or anticipatory SCR difference between CS+ vs. CS-, we performed a posterior-based association analysis inspired by the three-step approach of measuring the relationship between latent variables and covariates (Vermunt, 2010). After fitting the latent mixture model, we extracted the posterior probabilities of group membership for each participant $p_{z[i]}^{(s)}$, where s indexes posterior samples and i indexes participants. To assess the relationship between group membership and a covariate, we computed the Spearman rank-order correlation between the posterior probabilities of belonging to group 1 and the covariate values at each posterior draw:

$$r^{(s)} = \text{Spearman}(p_z^{(s)}, x)$$

where $p_z^{(s)} \in \mathbb{R}^n$ is the vector of probabilities indicating group 1 membership for all n participants at draw s and $x \in \mathbb{R}^n$ is the covariate vector. This yields a posterior distribution over the correlation coefficient r reflecting the uncertainty in both class membership and its association with the covariate.

Baseline two-high-threshold model (2HT): Gaussian Process extension

To explore whether individual differences in memory recognition vary systematically with subject-level features – assuming a single generative process shared across all participants – we extended the baseline 2HT model. Specifically, we placed a Gaussian Process (GP) prior over the

Figure S2

Baseline two-high-threshold model extended with Gaussian Process.

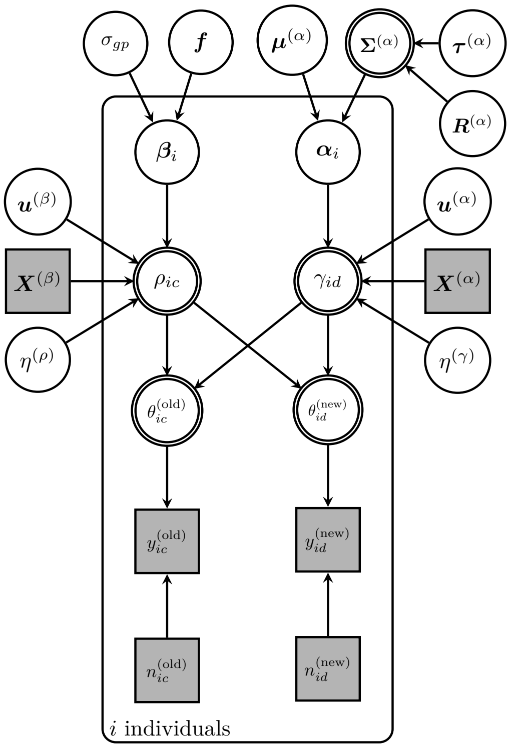

$$
\begin{aligned}
\ell &\sim \text{invGamma}(5,5)\\
\tau_{gp} &\sim \text{invGamma}(5,5)\\
k(x|\tau_{gp},\ell) &= \tau_{gp}\cdot\left(1+\tfrac{\sqrt{3}\|x-x'\|}{\ell}\right)\exp\left(-\tfrac{\sqrt{3}\|x-x'\|}{\ell}\right)\\
f_j(x) &\sim \text{MvNormal}(\mathbf{0}, k(x|\tau_{gp},\ell)),\ j\in\{1{:}6\}\\
\sigma_{gp} &\sim \text{Gamma}(5,10)\\
\mu_1^{(\alpha)} &\sim \text{Normal}(-0.8,1.5)\\
\mu_2^{(\alpha)} &\sim \text{Normal}(0,1)\\
\tau_j^{(\beta)} &\sim \text{Gamma}(5,10),\ j\in\{1{:}6\}\\
\tau_k^{(\alpha)} &\sim \text{Gamma}(5,10),\ k\in\{1,2\}\\
\boldsymbol{R}^{(\alpha)} &\sim \text{LKJcorr}(2),\ \boldsymbol{R}^{(\alpha)}\in\mathbb{R}^{2\times2}\\
\boldsymbol{\Sigma}^{(\alpha)} &= \text{diag}(\boldsymbol{\tau}^{(\alpha)})\cdot\boldsymbol{R}^{(\alpha)}\cdot\text{diag}(\boldsymbol{\tau}^{(\alpha)})\\
\boldsymbol{\beta}_i &\sim \text{MvNormal}(\boldsymbol{f}(x_i),\ \text{diag}(\sigma_{gp})^2),\ \boldsymbol{\beta}_i\in\mathbb{R}^{6\times1}\\
\boldsymbol{\alpha}_i &\sim \text{MvNormal}(\boldsymbol{\mu}^{(\alpha)},\ \boldsymbol{\Sigma}^{(\alpha)}),\ \boldsymbol{\alpha}_i\in\mathbb{R}^{2\times1}\\
\omega_j^{(\beta)},\ \omega_k^{(\alpha)} &\sim \text{Uniform}(0.01,0.4),\ j\in\{1{:}6\},\ k\in\{1,2\}\\
u_{j[study]}^{(\beta)} &\sim \text{Normal}(0,\omega_j^{(\beta)}),\ study\in\{1{:}4\}\\
u_{k[study]}^{(\alpha)} &\sim \text{Normal}(0,\omega_k^{(\alpha)}),\ study\in\{1{:}4\}\\
\boldsymbol{u}_{[study]}^{(\beta)},\ \boldsymbol{u}_{[study]}^{(\alpha)} &= [u_{1[study]}^{(\beta)},\dots,u_{6[study]}^{(\beta)}]^\top,\ [u_{1[study]}^{(\alpha)},u_{2[study]}^{(\alpha)}]^\top\\
c &= \begin{cases}1,2,3 & \text{for phases } 1\text{–}3^{(\text{CS}-)}\\ 4,5,6 & \text{for phases } 1\text{–}3^{(\text{CS}+)}\end{cases}\\
d &= 1 \text{ for CS}-,\ 2 \text{ for CS}+\\
w_c,\ w_d &= \begin{cases}-1/2 & \text{if } c,d\in\{\text{animals}\}\\ \ \ 1/2 & \text{if } c,d\in\{\text{tools}\}\end{cases}\\
\eta^{(\rho)},\ \eta^{(\gamma)} &\sim \text{Normal}(0,1)\\
\text{logit}(\rho_{ic}) &= \boldsymbol{X}_c^{(\beta)}\cdot(\boldsymbol{\beta}_i+\boldsymbol{u}_{[study]}^{(\beta)})+w_c\ \eta^{(\rho)},\ \boldsymbol{X}_c^{(\beta)}\in\mathbb{R}^{1\times6}\\
\text{logit}(\gamma_{id}) &= \boldsymbol{X}_d^{(\alpha)}\cdot(\boldsymbol{\alpha}_i+\boldsymbol{u}_{[study]}^{(\alpha)})+w_d\ \eta^{(\gamma)},\ \boldsymbol{X}_d^{(\alpha)}\in\mathbb{R}^{1\times2}\\
\theta_{ic}^{(\text{old})} &= \begin{cases}\rho_{ic}+(1-\rho_{ic})\ \gamma_{i1}, & c\in\{1,2,3\}\\ \rho_{ic}+(1-\rho_{ic})\ \gamma_{i2}, & c\in\{4,5,6\}\end{cases}\\
\theta_{id}^{(\text{new})} &= \begin{cases}(1-\rho_{i1}\ \rho_{i2}\ \rho_{i3})\ \gamma_{i1} & \text{if } d=1\\ (1-\rho_{i4}\ \rho_{i5}\ \rho_{i6})\ \gamma_{i2} & \text{if } d=2\end{cases}\\
y_{ic}^{(\text{old})} &\sim \text{Binomial}(\theta_{ic}^{(\text{old})},n_{ic}^{(\text{old})}),\ c\in\{1{:}6\}\\
y_{id}^{(\text{new})} &\sim \text{Binomial}(\theta_{id}^{(\text{new})},n_{id}^{(\text{new})}),\ d\in\{1,2\}
\end{aligned}
$$

Note. Graphical model of the extended 2HT model with a Gaussian Process (GP) prior over the memory recognition parameters, $\boldsymbol{\beta}_i$. Rather than sampling $\boldsymbol{\beta}_i$ directly from a multivariate normal distribution (as in the baseline model), this approach models them as noisy evaluations of a latent function $f(x)$ defined over subject-level covariates x (e.g., learning rate or anticipatory SCR). The GP captures smooth, nonlinear relationships between covariates and recognition performance. GP hyperparameters – kernel amplitude τ_{gp}, length scale ℓ, and observation noise σ_{gp} – are assigned weakly informative priors.

individual $\boldsymbol{\beta}_i \in \mathbb{R}^{6\times1}$ parameters, allowing them to flexibly vary as a function of covariates such as the RW learning rate and anticipatory physiological arousal (see graphical model in Figure S2).

In the baseline model, the subject-specific parameters $\boldsymbol{\beta}_i$ are drawn from a multivariate normal distribution with parameter-specific population means:

$$\boldsymbol{\beta}_i \sim \text{MvNormal}(\boldsymbol{\mu}^{(\beta)}, \boldsymbol{\Sigma}^{(\beta)}).$$

In the GP-extended model, we instead model these weights as noisy evaluations of latent functions defined over a covariate space:

$$\boldsymbol{\beta}_i \sim \text{MvNormal}(\boldsymbol{f}(x_i), \text{diag}(\sigma_{gp}^2)),$$

where $\boldsymbol{f}(x_i) \in \mathbb{R}^6$ denotes the vector of function outputs evaluated at the covariate vector

x_i for subject i, and σ^2_{gp} captures independent Gaussian observation noise for each parameter.

Each of the six functions in $\boldsymbol{f}$ is modeled as a draw from a zero-mean GP with a shared Matérn 3/2 kernel:

$$f_j(x) \sim \text{GP}(0,\ k(x, x' \mid \tau_{gp}, \ell)), \quad \text{for } j = 1, \ldots, 6,$$
$$k(x, x' \mid \tau_{gp}, \ell) = \tau_{gp} \cdot \left(1 + \frac{\sqrt{3}\|x - x'\|}{\ell}\right) \exp\left(-\frac{\sqrt{3}\|x - x'\|}{\ell}\right),$$

where τ_{gp} controls the output scale and ℓ the length scale. This kernel captures moderate smoothness and is computationally efficient, making it well suited for small- to medium-scale modeling.

The input features x_i varied across two model variants. In one, $x_i \in \mathbb{R}$ represented the subject's learning rate from the RW model. In the other, $x_i \in \mathbb{R}^2$ consisted of anticipatory SCR values derived from two distinct estimation methods.

We placed standard weakly informative priors on the GP hyperparameters:

$$\begin{aligned} \ell &\sim \text{InvGamma}(5, 5) \quad \text{(length scale)} \\ \tau_{gp} &\sim \text{InvGamma}(5, 5) \quad \text{(kernel amplitude)} \\ \sigma_{gp} &\sim \text{Gamma}(5, 10) \quad \text{(observation noise).} \end{aligned}$$

This GP-based framework allowed us to assess whether memory recognition varies systematically across a latent space defined by either individual learning dynamics or anticipatory physiological arousal.

Bayesian one-high-threshold (1HT) model of memory recognition

The one-high-threshold (1HT) recognition model shares the same underlying assumptions and decision-tree framework as the two-high-threshold (2HT) model, with a key distinction in the treatment of false alarms.

In the 2HT model, false alarms are attributed to a combination of detecting new (i.e., memory recognition) and guessing. In contrast, the 1HT model assumes that false alarms result exclusively from guessing, with no contribution from memory processes. Hit rates remain a mixture of memory recognition and guessing. This implies that, under the current design, responses to new items are not affected by memory of previously encoded items from the same semantic category.

As a result, the 1HT model adopts the same data-generating assumptions and prior distribution specifications as the 2HT model (see Figure S3 for a graphical representation), with the sole difference being in the modeling of false alarm rates. Specifically, false alarms are modeled using a binomial likelihood function, where the parameter $\theta^{(\text{new})}_{id}$ is set equal to the guessing parameter γ_{id}:

$$\theta^{(\text{new})}_{id} = \gamma_{id},$$

where d indicates semantic category assigned to either CS- or CS+ condition.

To evaluate model adequacy and potential mis-specification, we conducted posterior predictive checks. These included comparisons between observed and simulated data for summary statistics – specifically, grand means of hits and false alarms – aggregated both across and within studies.

Bayesian model of memory recognition based on signal detection theory (SDT)

The memory recognition model based on signal detection theory (SDT) belongs to a different family than threshold models, treating memory strength as a continuous variable. While threshold models assume categorical, all-or-none representations, SDT treats memory strength as continuous, with recognition occurring when strength exceeds a decision criterion.

Figure S3

Graphical model representation of the Bayesian one-high-threshold model for old–new recognition judgments.

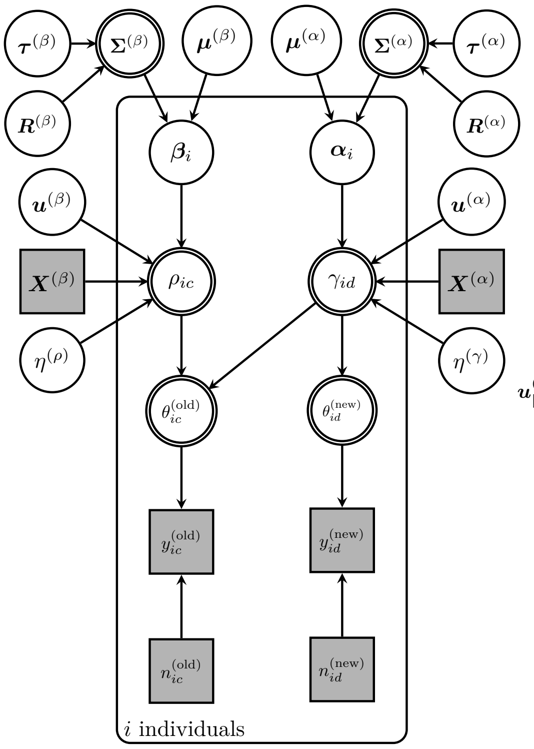

$$
\begin{aligned}
\mu_1^{(\beta)} &\sim \text{Normal}(-0.5, 1.5) \\
\mu_j^{(\beta)} &\sim \text{Normal}(0, 1),\ j \in \{2{:}6\} \\
\mu_1^{(\alpha)} &\sim \text{Normal}(-0.8, 1.5) \\
\mu_2^{(\alpha)} &\sim \text{Normal}(0, 1) \\
\tau_j^{(\beta)} &\sim \text{Gamma}(5, 10),\ j \in \{1{:}6\} \\
\tau_k^{(\alpha)} &\sim \text{Gamma}(5, 10),\ k \in \{1, 2\} \\
\boldsymbol{R}^{(\beta)} &\sim \text{LKJcorr}(2),\ \boldsymbol{R}^{(\beta)} \in \mathbb{R}^{6\times 6} \\
\boldsymbol{R}^{(\alpha)} &\sim \text{LKJcorr}(2),\ \boldsymbol{R}^{(\alpha)} \in \mathbb{R}^{2\times 2}
\end{aligned}
$$

$$
\begin{aligned}
\boldsymbol{\Sigma}^{(\beta)} &= \text{diag}(\boldsymbol{\tau}^{(\beta)}) \cdot \boldsymbol{R}^{(\beta)} \cdot \text{diag}(\boldsymbol{\tau}^{(\beta)}) \\
\boldsymbol{\Sigma}^{(\alpha)} &= \text{diag}(\boldsymbol{\tau}^{(\alpha)}) \cdot \boldsymbol{R}^{(\alpha)} \cdot \text{diag}(\boldsymbol{\tau}^{(\alpha)})
\end{aligned}
$$

$$
\begin{aligned}
\boldsymbol{\beta}_i &\sim \text{MvNormal}(\boldsymbol{\mu}^{(\beta)},\ \boldsymbol{\Sigma}^{(\beta)}),\ \boldsymbol{\beta}_i \in \mathbb{R}^{6\times 1} \\
\boldsymbol{\alpha}_i &\sim \text{MvNormal}(\boldsymbol{\mu}^{(\alpha)},\ \boldsymbol{\Sigma}^{(\alpha)}),\ \boldsymbol{\alpha}_i \in \mathbb{R}^{2\times 1}
\end{aligned}
$$

$$
\begin{aligned}
\omega_j^{(\beta)},\ \omega_k^{(\alpha)} &\sim \text{Uniform}(0.01, 0.4),\ j \in \{1{:}6\},\ k \in \{1, 2\} \\
u_{j[study]}^{(\beta)} &\sim \text{Normal}(0, \omega_j^{(\beta)}),\ study \in \{1{:}4\} \\
u_{k[study]}^{(\alpha)} &\sim \text{Normal}(0, \omega_k^{(\alpha)}),\ study \in \{1{:}4\} \\
\boldsymbol{u}_{[study]}^{(\beta)},\ \boldsymbol{u}_{[study]}^{(\alpha)} &= [u_{1[study]}^{(\beta)}, \ldots, u_{6[study]}^{(\beta)}]^\top,\ [u_{1[study]}^{(\alpha)}, u_{2[study]}^{(\alpha)}]^\top
\end{aligned}
$$

$$
\begin{aligned}
c &= \begin{cases} 1, 2, 3 & \text{for phases 1–3}^{(\text{CS}-)} \\ 4, 5, 6 & \text{for phases 1–3}^{(\text{CS}+)} \end{cases} \\
d &= 1 \text{ for CS}-,\ 2 \text{ for CS}+ \\
w_c,\ w_d &= \begin{cases} -1/2 & \text{if } c, d \in \{\text{animals}\} \\ \ \ 1/2 & \text{if } c, d \in \{\text{tools}\} \end{cases}
\end{aligned}
$$

$$
\eta^{(\rho)},\ \eta^{(\gamma)} \sim \text{Normal}(0, 1)
$$

$$
\begin{aligned}
\text{logit}(\rho_{ic}) &= \boldsymbol{X}_c^{(\beta)} \cdot (\boldsymbol{\beta}_i + \boldsymbol{u}_{[study]}^{(\beta)}) + w_c\ \eta^{(\rho)},\ \boldsymbol{X}_c^{(\beta)} \in \mathbb{R}^{1\times 6} \\
\text{logit}(\gamma_{id}) &= \boldsymbol{X}_d^{(\alpha)} \cdot (\boldsymbol{\alpha}_i + \boldsymbol{u}_{[study]}^{(\alpha)}) + w_d\ \eta^{(\gamma)},\ \boldsymbol{X}_d^{(\alpha)} \in \mathbb{R}^{1\times 2}
\end{aligned}
$$

$$
\begin{aligned}
\theta_{ic}^{(\text{old})} &= \begin{cases} \rho_{ic} + (1 - \rho_{ic})\ \gamma_{i1}, & c \in \{1, 2, 3\} \\ \rho_{ic} + (1 - \rho_{ic})\ \gamma_{i2}, & c \in \{4, 5, 6\} \end{cases} \\
\theta_{id}^{(\text{new})} &= \gamma_{id}
\end{aligned}
$$

$$
\begin{aligned}
y_{ic}^{(\text{old})} &\sim \text{Binomial}(\theta_{ic}^{(\text{old})}, n_{ic}^{(\text{old})}),\ c \in \{1{:}6\} \\
y_{id}^{(\text{new})} &\sim \text{Binomial}(\theta_{id}^{(\text{new})}, n_{id}^{(\text{new})}),\ d \in \{1, 2\}
\end{aligned}
$$

Note. Unlike the 2HT model, where false alarms arise from memory recognition and guessing, this model assumes false alarms arise solely from guessing, excluding any recognition influence.

SDT assumes two overlapping normal distributions with equal variance: one representing *noise* (new items) and the other representing *signal* (old items). An individual evaluates each item and makes a recognition decision based on whether its memory strength surpasses a predefined criterion.

Two key parameters define performance in this model. Sensitivity (d') measures the ability to discriminate between old (signal) and new (noise) items with higher values indicating better discrimination. Criterion (λ) reflects the decision threshold or bias. It determines how liberal or conservative the participant is when deciding whether an item is *old*. For instance, a liberal criterion yields more hits but also more false alarms. Because there is only one false alarm rate per semantic category in the experimental design, SDT assumes a consistent criterion across all items within that category, regardless of encoding phase. This simplifying assumption, though often reasonable, may conflate true differences in discriminability with unmodeled shifts in response bias. Such shifts may arise if participants adjust caution based on perceived memory strength or source (e.g., being more liberal / conservative for items believed to be shown during conditioning) or base-rate beliefs (e.g., adopting a stricter / weaker criterion if more trials are believed to be shown during conditioning). In such cases, what appears to be a difference in discriminability might instead reflect a change in bias.

Figure S4

Graphical model representation of the Bayesian model for old–new recognition judgments based on signal detection theory.

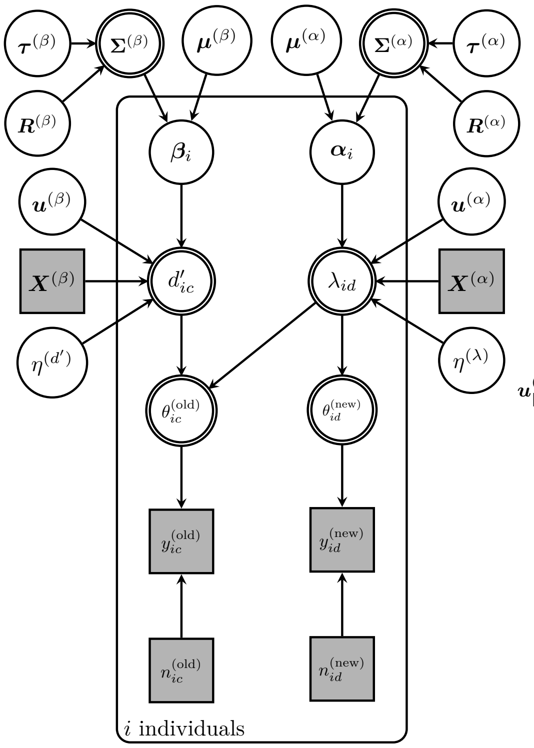

$$
\begin{aligned}
\mu_1^{(\beta)} &\sim \text{Normal}(1, 0.5)\\
\mu_j^{(\beta)} &\sim \text{Normal}(0, 0.5),\ j \in \{2{:}6\}\\
\mu_1^{(\alpha)} &\sim \text{Normal}(0.5, 0.5)\\
\mu_2^{(\alpha)} &\sim \text{Normal}(0, 0.5)\\
\tau_j^{(\beta)} &\sim \text{Gamma}(5, 10),\ j \in \{1{:}6\}\\
\tau_k^{(\alpha)} &\sim \text{Gamma}(5, 10),\ k \in \{1, 2\}\\
\boldsymbol{R}^{(\beta)} &\sim \text{LKJcorr}(2),\ \boldsymbol{R}^{(\beta)} \in \mathbb{R}^{6\times 6}\\
\boldsymbol{R}^{(\alpha)} &\sim \text{LKJcorr}(2),\ \boldsymbol{R}^{(\alpha)} \in \mathbb{R}^{2\times 2}\\
\boldsymbol{\Sigma}^{(\beta)} &= \text{diag}(\boldsymbol{\tau}^{(\beta)}) \cdot \boldsymbol{R}^{(\beta)} \cdot \text{diag}(\boldsymbol{\tau}^{(\beta)})\\
\boldsymbol{\Sigma}^{(\alpha)} &= \text{diag}(\boldsymbol{\tau}^{(\alpha)}) \cdot \boldsymbol{R}^{(\alpha)} \cdot \text{diag}(\boldsymbol{\tau}^{(\alpha)})\\
\boldsymbol{\beta}_i &\sim \text{MvNormal}(\boldsymbol{\mu}^{(\beta)},\ \boldsymbol{\Sigma}^{(\beta)}),\ \boldsymbol{\beta}_i \in \mathbb{R}^{6\times 1}\\
\boldsymbol{\alpha}_i &\sim \text{MvNormal}(\boldsymbol{\mu}^{(\alpha)},\ \boldsymbol{\Sigma}^{(\alpha)}),\ \boldsymbol{\alpha}_i \in \mathbb{R}^{2\times 1}\\
\omega_j^{(\beta)},\ \omega_k^{(\alpha)} &\sim \text{Uniform}(0.01, 0.3),\ j \in \{1{:}6\},\ k \in \{1, 2\}\\
u_{j[study]}^{(\beta)} &\sim \text{Normal}(0, \omega_j^{(\beta)}),\ study \in \{1{:}4\}\\
u_{k[study]}^{(\alpha)} &\sim \text{Normal}(0, \omega_k^{(\alpha)}),\ study \in \{1{:}4\}\\
\boldsymbol{u}_{[study]}^{(\beta)},\ \boldsymbol{u}_{[study]}^{(\alpha)} &= [u_{1[study]}^{(\beta)}, \ldots, u_{6[study]}^{(\beta)}]^\top,\ [u_{1[study]}^{(\alpha)}, u_{2[study]}^{(\alpha)}]^\top\\
c &= \begin{cases} 1, 2, 3 & \text{for phases 1–3}^{(\text{CS}-)} \\ 4, 5, 6 & \text{for phases 1–3}^{(\text{CS}+)} \end{cases}\\
d &= 1 \text{ for CS}-,\ 2 \text{ for CS}+\\
w_c,\ w_d &= \begin{cases} -1/2 & \text{if } c, d \in \{\text{animals}\} \\ 1/2 & \text{if } c, d \in \{\text{tools}\} \end{cases}\\
\eta^{(d')},\ \eta^{(\lambda)} &\sim \text{Normal}(0, 0.5)\\
\text{logit}(d'_{ic}) &= \boldsymbol{X}_c^{(\beta)} \cdot (\boldsymbol{\beta}_i + \boldsymbol{u}_{[study]}^{(\beta)}) + w_c\, \eta^{(d')},\ \boldsymbol{X}_c^{(\beta)} \in \mathbb{R}^{1\times 6}\\
\text{logit}(\lambda_{id}) &= \boldsymbol{X}_d^{(\alpha)} \cdot (\boldsymbol{\alpha}_i + \boldsymbol{u}_{[study]}^{(\alpha)}) + w_d\, \eta^{(\lambda)},\ \boldsymbol{X}_d^{(\alpha)} \in \mathbb{R}^{1\times 2}\\
\theta_{ic}^{(\text{old})} &= \begin{cases} \Phi(d'_{ic} - \lambda_{i1}), & c \in \{1, 2, 3\} \\ \Phi(d'_{ic} - \lambda_{i2}), & c \in \{4, 5, 6\} \end{cases}\\
\theta_{id}^{(\text{new})} &= \Phi(-\lambda_{id})\\
y_{ic}^{(\text{old})} &\sim \text{Binomial}(\theta_{ic}^{(\text{old})}, n_{ic}^{(\text{old})}),\ c \in \{1{:}6\}\\
y_{id}^{(\text{new})} &\sim \text{Binomial}(\theta_{id}^{(\text{new})}, n_{id}^{(\text{new})}),\ d \in \{1, 2\}
\end{aligned}
$$

Note. Sensitivity (d') measures the ability to distinguish old from new items, while the criterion (λ) reflects the decision threshold.

A graphical representation of the model, including the assumptions about the data-generating process and the specification of prior distributions, is shown in Figure S4. The overall structure follows the 1HT model, with key modifications in the modeling of hits and false alarms, and in the choice of a few prior distributions. Notably, the model departs from the log-odds space and instead operates in z-units, consistent with the assumption of both the signal and noise distributions to be the standard normal distributions.

The model predicts the number of *hits*, $y_{ic}^{(\text{old})}$, out of $n_{ic}^{(\text{old})}$ trials, with i indexes participants and c indexes the six experimental conditions using a binomial likelihood function with the success probability parameter $\theta_{ic}^{(\text{old})}$, which is defined according to the equation:

$$
\theta_{ic}^{(\text{old})} = \begin{cases} \Phi(d'_{ic} - \lambda_{i1}), & \text{when } c \in \text{CS}- \\ \Phi(d'_{ic} - \lambda_{i2}), & \text{when } c \in \text{CS}+, \end{cases}
$$

where Φ denotes the cumulative distribution function of the standard normal distribution. The sensitivity parameter d'_{ic} is condition- and participant-specific, while the criterion λ_{id} varies by semantic category (i.e., the semantic category assigned to CS+ vs. CS-) but is held constant across experimental phases (pre-conditioning, conditioning, and post-conditioning).

The hierarchical structure mirrors that of the 1HT and 2HT models. The population-level prior for the grand mean sensitivity parameter across conditions, $\mu_1^{(\beta)}$ was adapted to the z-unit scale and chosen based on prior predictive checks:

$$\mu_1^{(\beta)} \sim \text{Normal}(1, 0.5).$$

The model also predicts the number of *false alarms*, $y_{id}^{(\text{new})}$, out of $n_{id}^{(\text{new})}$ trials, where i indexes participants and d indexes the two semantic categories allocated to CS- and CS+ conditions, using a binomial likelihood function. The success probability parameter $\theta_{id}^{(\text{new})}$ is defined according to the equation:

$$\theta_i^{(\text{new})} = \begin{cases} \Phi(-\lambda_{i1}), & \text{for CS-} \\ \Phi(-\lambda_{i2}) & \text{for CS+}. \end{cases}$$

The population-level prior for the grand mean of the bias (i.e., response criterion), $\mu_1^{(\alpha)}$, was also adapted to z-units based on prior predictive checks:

$$\mu_1^{(\alpha)} \sim \text{Normal}(0.5, 0.5).$$

To evaluate model adequacy and potential mis-specification, we conducted posterior predictive checks. These included comparisons between observed and simulated data for summary statistics – specifically, grand means of hits and false alarms – aggregated both across and within studies.

Cross-validated comparison of baseline models: 1HT, 2HT, and SDT

We compared the three baseline models (1HT, 2HT, and SDT) using 5-fold cross-validation stratified by subject and study. Each model was trained on 80% of the data and tested on the remaining 20%, with out-of-sample predictive performance measured via expected log predictive density (ELPD) via the `loo` R package (Vehtari et al., 2024). Comparisons were based on differences in ELPD, with higher values indicating better fit; following common guidelines (Vehtari et al., 2016), differences exceeding twice their standard error were considered meaningful.

Supplementary Results

Reproducibility checks

The validity of the retrieved data was successfully verified. Visual inspection revealed that the data are free from errors or inconsistencies (Figure S5). Likewise, we successfully reproduced the main results of the original report (Figure S6).

Latent-mixture two-high-threshold model (2HT): Parameter replicability across independent datasets

To assess whether the latent subgroup structure identified by the mixture 2HT model is reproducible across independent datasets, we fit the model separately to each of the three larger studies and quantified the consistency of the resulting parameter estimates. Posterior similarity was evaluated using a density-overlap metric (κ) computed pairwise across studies for all parameters of interest (Figure S7). This analysis provides a direct test of whether the latent components reflect stable structure in the data or whether they might instead arise from study-specific noise or sampling variability.

The mixture weight parameter ϕ showed relatively strong agreement ($\kappa \approx 0.77$). Among the regression coefficients, overlap estimates ranged from ≈ 0.47 to 0.80, indicating that some parameters were estimated more reliably across independent datasets than others. Specifically, parameters such as β_3 (Δ phase 3 vs. phase 2), β_4 (Δ CS+ vs. CS- in pre-conditioning), and β_5 (Δ CS+ vs. CS- in conditioning) in group 1, as well as β_3 (Δ phase 3 vs. phase 2) in group 2

Figure S5

Raw old-new recognition judgments of all participants across four studies.

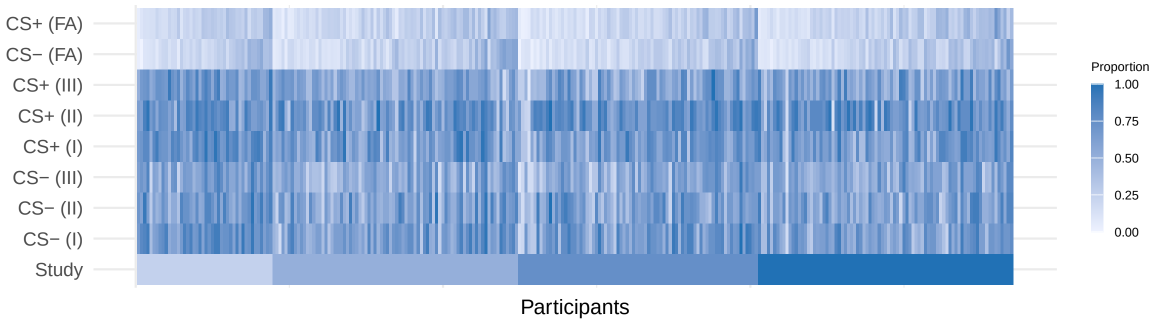

Note. Raw hit rates and false alarms per participant. Roman numbers indicate phase of the encoding: preconditioning (I), conditioning (II), and post-conditioning (II). Last row indicates the study id that is color-coded.

displayed high agreement ($\kappa \approx$ 0.71–0.80). Other parameters such as β_1 (grand mean), β_6 (Δ CS+ vs. CS- in post-conditioning) in group 1, as well as β_2 (Δ phase 2 vs. phase 1), β_4 (Δ CS+ vs. CS- in pre-conditioning), and β_6 (Δ CS+ vs. CS- in post-conditioning) in group 2 exhibited moderately stable parameters ($\kappa \approx$ 0.53–0.67). Finally, the other parameters: β_2 (Δ phase 2 vs. phase 1) in group 1, as well as β_2 (Δ phase 2 vs. phase 1) and β_5 (Δ CS+ vs. CS- in conditioning) in group 2 are the least stable. For mixture models fitted to datasets of ~80 participants, overlap values around 0.45–0.60 are typical.

Overall, the level of consistency observed across datasets suggests that the mixture structure is supported by the data and that the key inferential patterns are robust, ruling out the possibility that the latent components are driven by procedural differences between studies.

Latent-mixture two-high-threshold model (2HT): Posterior group membership across studies

To complement the independent-dataset analysis, we next examined whether the two latent groups identified by the mixture model were consistently represented across studies when the model was fit to the full pooled dataset. This analysis assesses whether both latent subpopulations appear across studies under a single shared parameterization, and whether group assignments exhibit similar degrees of separation.

The prevalence of the first group was generally consistent across studies, with Study 1 showing the lowest proportion (Study 1: 0.32, Study 2: 0.44, Study 3: 0.49, Study 4: 0.42).

Visual inspection of ECDFs confirmed the presence of a bimodal distribution in posterior group membership probabilities across all studies, indicating that both groups are represented within each study. To quantify between-study differences in group membership distributions, we computed the Kolmogorov–Smirnov (KS) distance between the ECDFs at each MCMC draw, subsampling larger studies to match the size of the smaller ones. Distances ranged from small to moderate. Study 1 differed most from the others (study 1 vs. study 2: KS = 0.19, 89% HPDI [0.11, 0.3]; study 1 vs. study 3: KS = 0.23, 89% HPDI [0.11, 0.34]; study 1 vs. study 4: KS = 0.2, 89% HPDI [0.11,

Figure S6

Reproducibility outcome comparing reported and reproduced values of statistical analyses.

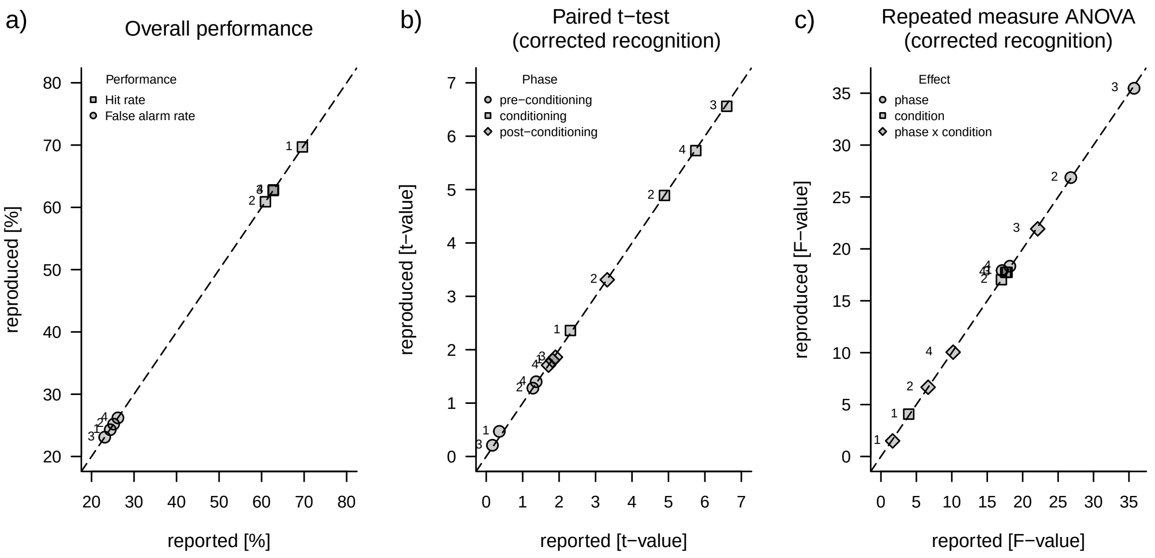

Note. The dashed line is an identity line (x = y). Numbers above the data points indicate study id.

0.3]). In contrast, the distributions from Study 2 and Study 4 were highly similar, with minimal divergence (KS = 0.11, 89% HPDI [0.06, 0.16]).

These findings indicate that, despite some variability – particularly Study 1 – the two latent groups are robustly recovered when the model is fit to the pooled dataset, and no study is associated exclusively with one group.

Gaussian Process extension of two-high-threshold model (2HT)

We explored whether individual differences in Pavlovian learning contribute to recognition memory performance using the 2HT model extended with a Gaussian Process (GP).

Learning rate

First, we considered the role of the α learning rate of the RW model (Figure S8), which reflects how quickly individuals update the associative strength between a semantic category and the electric shock. This metric essentially serves as an index of how effectively participants distinguish between the two categories.

The estimated length scale parameter was relatively high (l = 3.28, 89% HPDI [1.63, 5.89]), indicating that the underlying functions were fairly smooth, with minimal variation in response to small changes in the learning rate. The GP accounted for a moderate level of noise in the participant-level recognition parameters (σ_{gp} = 0.6, 89% HPDI [0.56, 0.64]), suggesting the presence of some process noise.

When examining individual profiles of GP latent functions, we observed a strong divergence in selective memory prioritization for items encoded during conditioning (CS+ vs. CS-) between participants with low vs. high learning rates. Specifically, individuals who rapidly learned the category-shock association exhibited a clear selective memory advantage for CS+ items over CS- items (i.e., the latent function lies outside the ROPE). In contrast, for individuals with slower learning rates, this effect was unreliable, with the latent function primarily overlapping with ROPE.

A similar pattern emerged when comparing recognition memory across encoding phases. Individuals with faster learning rates showed better recognition for items encoded during conditioning

Figure S7

Parameter replicability of the latent-mixture 2HT model across independent datasets.

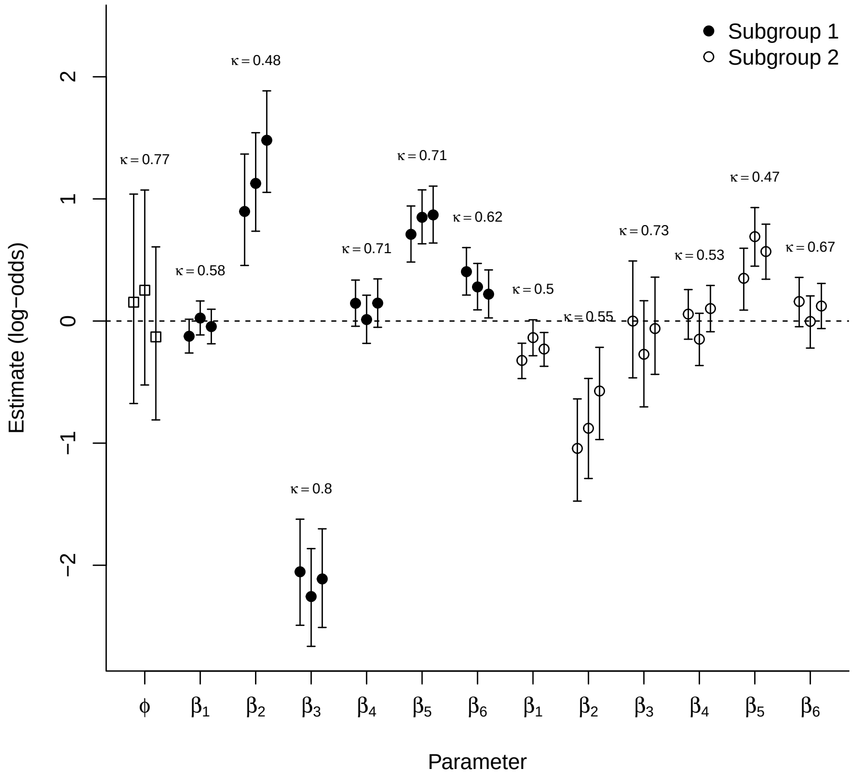

Note. Posterior estimates with 89% HPDI for all parameters of interest are shown for each of the three larger studies, with the mixture 2HT model fit separately to each dataset. The value above each parameter indicates the average pairwise posterior overlap (κ) across studies, which quantifies distributional similarity on a scale from 0 (no overlap) to 1 (complete overlap). Higher values therefore reflect greater replicability of the parameter estimates across independent datasets. Together, the pattern of overlapping posterior densities and κ values provides a direct assessment of the stability of the subgroup-defining effects and evaluates whether the latent mixture structure is reproducible across studies rather than driven by study-specific variability.

– regardless of semantic category – paired with lower recognition for items encoded post-conditioning. The reverse was true for slower learners.

Additionally, we observed a qualitative linear trend in selective memory prioritization for items encoded during post-conditioning as a function of learning rate: as the learning rate increased, the latent function progressively moved away from ROPE. However, due to high uncertainty in the estimates, these observations remain inconclusive.

Overall, beyond these specific effects, the learning rate showed limited relevance for explaining broader individual differences in recognition memory performance.

Differential anticipatory SCRs

The estimated length scale parameter was very high ($l = 6.43$, 89% HPDI [3.19, 12.26]), indicating that the underlying functions were very smooth, or perhaps even resembling a straight

Figure S8

Results of the Gaussian Process extension of two-high-threshold model (2HT) considering learning rate.

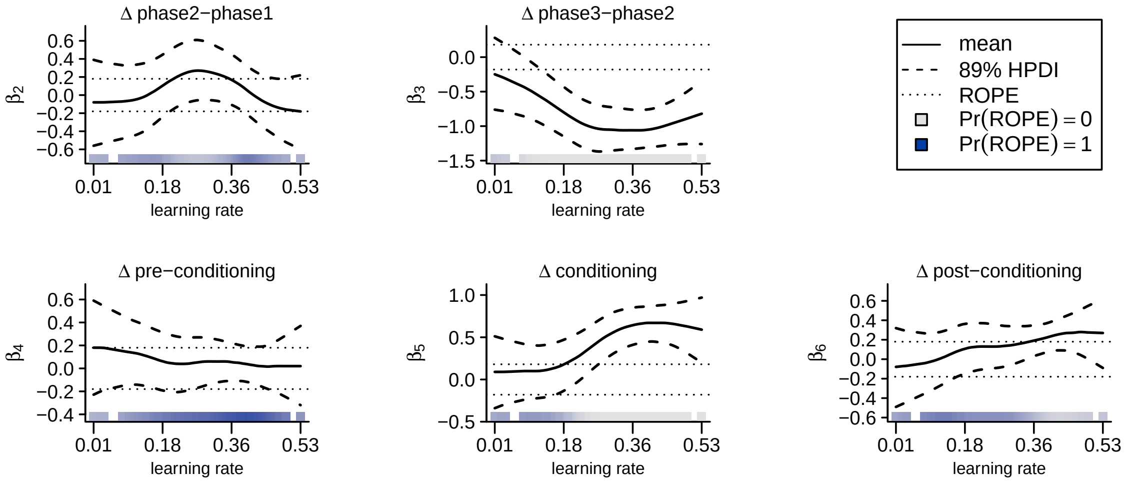

Note. Plots depict latent functions that map input features (learning rate) onto $\boldsymbol{\beta}_i$ parameters (e.g., CS+ vs. CS- difference nested within phases). Values of learning rate are bounded between 0 and 1. Pr_{ROPE} indicates the proportion of the posterior at a particular value of the learning rate that falls within the region of practical equivalence (+/-0.18 on the log-odds scale). Pr_{ROPE} is represented by color, ranging from gray (0) to dark blue (1).

line with minimal variation in response to big changes over input space. Similarly to the GP with learning rate, the model accounted for a moderate level of noise in the participant-level recognition parameters (σ_{gp} = 0.59, 89% HPDI [0.55, 0.63]), suggesting the presence of some process noise.

When considering the individual profiles of latent functions over the differential anticipatory SCRs, we observed no clear pattern except the category-selective memory prioritization effect for items encoded during conditioning (Figure S9). Medium-size difference in SCR corresponded with a clear effect (i.e., the latent function outside ROPE). These individuals also showed a reduced uncertainty in the putative effect of selective memory prioritization for items encoded in the post-conditioning phase.

Together, SCR proved to be only partially relevant in explaining selective memory prioritization for items encoded during conditioning, and it could not account for why the effect might appear in the pre- or post-conditioning phases.

Results of the one-high-threshold model (1HT)

The results of the Bayesian 1HT model largely mirrored those of the 2HT model (Figure S10), with only minor discrepancies – slightly higher raw recognition estimates and stronger participant-level correlations of random effects.

Specifically, the 1HT model produced slightly higher raw recognition estimates (~0.52 vs. ~0.49 in the 2HT model). Similar to the 2HT results, we observed a clear memory prioritization effect for CS+ items encoded during conditioning (OR = 1.88, 89% HPDI [1.55, 2.24], Pr_{ROPE} = .00), with higher recognition for CS+ (ρ = .64, 89% HPDI [.58, .69]) than CS- items (ρ = .49, 89% HPDI [.43, .55]). Recognition rates for items encoded during pre-conditioning were comparable across categories (ρ_{CS+} = .57, 89% HPDI [.51, .62]; ρ_{CS-} = .54, 89% HPDI [.49, .6]; OR = 1.1, 89% HPDI [0.96, 1.25], Pr_{ROPE} = .87), whereas post-conditioning recognition was slightly higher for CS+ items (ρ_{CS+} = .47, 89% HPDI [.41, .52]; ρ_{CS-} = .41, 89% HPDI [.35, .46]; OR = 1.28, 89% HPDI [1.13, 1.45], Pr_{ROPE} = .18), suggesting tentative evidence for selective and

Figure S9

Results of the Gaussian Process extension of two-high-threshold model (2HT) considering differential anticipatory SCR.

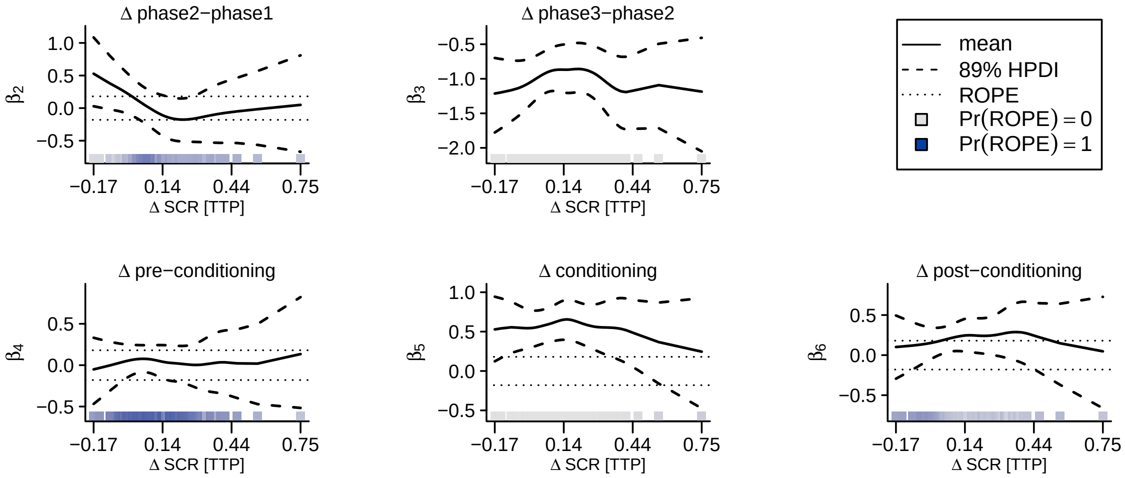

Note. Plots depict latent functions that map input features (CS+ vs. CS- difference in anticipatory SCR estimated with through-to-peak (TTP) method) onto β_i parameters (e.g., CS+ vs. CS- difference nested within phases). Values of ΔSCR are unbounded. Pr_{ROPE} indicates the proportion of the posterior at a particular value of the ΔSCR that falls within the region of practical equivalence (+/-0.18 on the log-odds scale). Pr_{ROPE} is represented by color, ranging from gray (0) to dark blue (1).

Figure S10

Results of the Bayesian 1HT model of old-new recognition judgments.

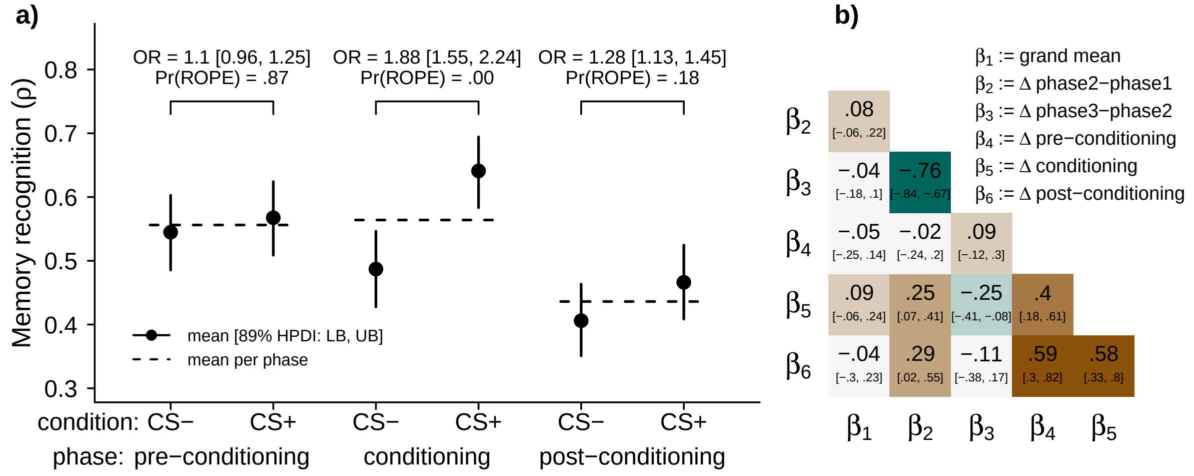

Note. Panel a depicts posterior estimates of memory recognition (ρ). Square brackets indicate 89% HPDI interval of odds ratio (OR). Proportion of the posterior falling inside the region of practical equivalence is denoted by Pr(ROPE). Panel b depicts within-subject correlation matrix of random effects (mean [89% HPDI: LB, UB]).

proactive memory prioritization – consistent with the 2HT model's findings.

The overall correlation structure among participant-level random effects was somewhat stronger in the 1HT model than in the 2HT model. We observed positive correlations in selec-

tive memory prioritization across phases: pre-conditioning and post-conditioning (r = .59, 89% HPDI [.3, .82], Pr_{ROPE} = .01), conditioning and post-conditioning (r = .58, 89% HPDI [.33, .8], Pr_{ROPE} = .00), as well as, pre-conditioning and conditioning (r = .4, 89% HPDI [.18, .61], Pr_{ROPE} = .02). These results suggest that – more so than in the 2HT model – some individuals consistently prioritized memory for CS+ items across all encoding phases, relative to the group average.

Results of model based on signal detection theory (SDT)

Figure S11

Results of the Bayesian SDT model of old-new recognition judgments.

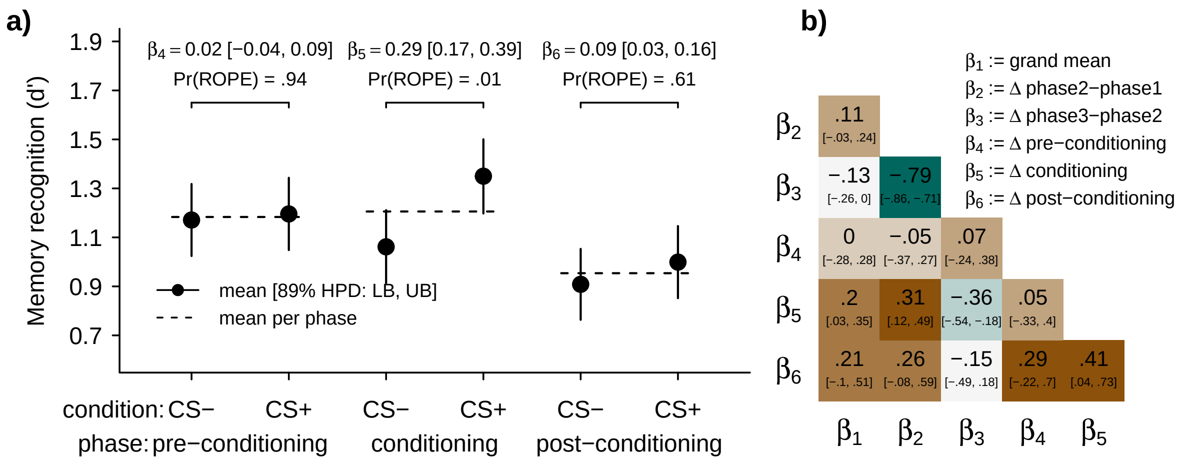

Note. Panel a depicts posterior estimates of sensitivity (d'). Square brackets indicate 89% HPDI interval. Proportion of the posterior falling inside the region of practical equivalence is denoted by Pr(ROPE), which is -0.1 and 0.1. Panel b depicts within-subject correlation matrix of random effects (mean [89% HPDI: LB, UB]).

The results of the Bayesian SDT model largely mirrored the qualitative pattern observed with the 2HT model (Figure S11), although the effect sizes were somewhat attenuated. To determine the presence of an effect in the sensitivity estimates (d'), we used a ROPE with a range of [-0.1, 0.1].

As in the other two models, we observed a clear memory prioritization effect for CS+ items encoded during conditioning ($\Delta\beta = 0.29$, 89% HPDI [0.17, 0.39], Pr_{ROPE} = .01), with higher recognition for CS+ ($d' = 1.35$, 89% HPDI [1.2, 1.5]) than CS- items ($d' = 1.06$, 89% HPDI [.91, 1.21]). Sensitivity values for items encoded during pre-conditioning were comparable across categories ($d'_{CS+} = 1.2$, 89% HPDI [1.05, 1.34]; $d'_{CS-} = 1.17$, 89% HPDI [1.02, 1.32]; $\Delta\beta = 0.02$, 89% HPDI [-0.04, 0.09], Pr_{ROPE} = .94), and post-conditioning recognition was slightly higher for CS+ items ($d'_{CS+} = 1$, 89% HPDI [.85, 1.15]; $d'_{CS-} = .91$, 89% HPDI [.76, 1.05]; $\Delta\beta = 0.09$, 89% HPDI [0.03, 0.16], Pr_{ROPE} = .61), but this difference largely fell within the ROPE, indicating little evidence for a meaningful effect. Although the post-conditioning effect had a lower probability of being distinct from zero than in the other models, it still showed a clearly positive direction.

The overall correlation structure among participant-level random effects was weaker in the SDT model than in the 1HT model. Correlations in selective memory prioritization across phases were generally small or uncertain: pre-conditioning and conditioning (r = .05, 89% HPDI [-0.33, .4], Pr_{ROPE} = .32), pre-conditioning and post-conditioning (r = .29, 89% HPDI [-0.22, .7], Pr_{ROPE} = .14), and conditioning and post-conditioning phases (r = .41, 89% HPDI [.04, .73], Pr_{ROPE} = .06). These results suggest little consistent relationship in selective memory prioritization across phases in the SDT model, with the possible exception of a moderate association between conditioning

and post-conditioning. Finally, we observed a strong negative correlation between phase-to-phase differences, regardless of semantic category (r = -0.79, 89% HPDI [-0.86, -0.71], Pr_{ROPE} = .00) that was also clearly present in other two models.

Results of baseline models comparison: 1HT, 2HT, and SDT

The ELPD-based comparison of predictive performance among the three baseline models ranked model fit as follows: 1HT (-9252.43), 2HT (-9260.19), and then SDT (-9296.55), with differences not large enough to distinguish between models (2HT vs. 1HT: ΔELPD = 7.76, SE = 12.08, z = 0.64; 2HT vs. SDT: ΔELPD = 36.36, SE = 24.4, z = 1.49), although the evidence marginally favored the 1HT model over SDT (ΔELPD = 44.12, SE = 23.15, z = 1.91).

This is not unexpected: all three models are fit to the same hit and false-alarm rates, which provide limited leverage for distinguishing among the models. In such settings, cross-validated predictive performance is not a sensitive criterion for model comparison, and the data are not well suited for decisive model selection. Our goal was therefore not to select the "best" baseline model, but to contextualize the 2HT framework relative to plausible alternatives.

Our retention of the 2HT model rests on theoretical and interpretive considerations that are essential for our individual-differences analyses. Although the baseline models achieve comparable predictive fits, they differ in how they treat false alarms and in the kinds of memory inferences they support. 1HT and SDT are limited in different ways.

In 1HT, false alarms arise solely from guessing and therefore contain no memory signal. As a result, false-alarm rates cannot inform estimates of phase-specific or individual-level memory variation.

In SDT, all phases necessarily share a single false-alarm process and decision criterion. This forces phase-specific discriminability estimates to depend on a common bias parameter. Because differences in accuracy across phases may reflect shifts in response bias rather than memory, SDT cannot yield identifiable phase-specific memory parameters independent of criterion variability.

The 2HT model addresses both limitations. By linking hits and false alarms through the same phase-specific detection processes, 2HT treats false alarms as a multi-phase detection failure rather than as pure guesses (1HT) or a fixed criterion-driven responses (SDT). This structure allows the single category-level false-alarm rate to be meaningfully related to the three phase-specific hit rates, yielding identifiable and interpretable estimates of memory variation across phases and individuals. As a result, the 2HT model yields more conservative estimates at the cost of slightly reduced predictive flexibility. For these reasons, 2HT provides the most suitable theoretical foundation for the individual-differences and mixture-model analyses conducted here.

References

Erdfelder, E., Auer, T.-S., Hilbig, B. E., Aßfalg, A., Moshagen, M., & Nadarevic, L. (2009). Multinomial processing tree models: A review of the literature. *Zeitschrift Für Psychologie / Journal of Psychology*, *217*(3), 108–124. https://doi.org/10.1027/0044-3409.217.3.108

Kalbe, F., & Schwabe, L. (2022). On the search for a selective and retroactive strengthening of memory: Is there evidence for category-specific behavioral tagging? *Journal of Experimental Psychology. General*, *151*(1), 263–284. https://doi.org/10.1037/xge0001075

Rescorla, R. A., & Wagner, A. R. (1972). A theory of Pavlovian conditioning: Variations on the effectiveness of reinforcement and non-reinforcement. In A. H. Black & W. F. Prokasy (Eds.), *Classical conditioning II: Current research and theory* (pp. 64–99). Appleton-Century-Crofts.

Snodgrass, J. G., & Corwin, J. (1988). Pragmatics of measuring recognition memory: Applications to dementia and amnesia. *Journal of Experimental Psychology: General*, *117*(1), 34–50. https://doi.org/10.1037/0096-3445.117.1.34

Stan Development Team. (2023). *Stan modeling language users guide and reference manual*. Stan Development Team. https://mc-stan.org

Stan Development Team. (2024). *RStan: The R interface to Stan*. https://mc-stan.org/

Tzovara, A., Korn, C. W., & Bach, D. R. (2018). Human pavlovian fear conditioning conforms to probabilistic learning. *PLOS Computational Biology*, *14*(8), e1006243. https://doi.org/10.1371/journal.pcbi.1006243

Vehtari, A., Gabry, J., Magnusson, M., Yao, Y., Bürkner, P.-C., Paananen, T., & Gelman, A. (2024). *Loo: Efficient leave-one-out cross-validation and WAIC for bayesian models*. https://mc-stan.org/loo/

Vehtari, A., Gelman, A., & Gabry, J. (2016). Practical bayesian model evaluation using leave-one-out cross-validation and WAIC. *Statistics and Computing*, *27*(5), 1413–1432. https://doi.org/10.1007/s11222-016-9696-4

Vermunt, J. K. (2010). Latent class modeling with covariates: Two improved three-step approaches. *Political Analysis*, *18*(4), 450–469. http://www.jstor.org/stable/25792024

Appendix

Appendix A: Relationship between the two-high-threshold (2HT) model and the corrected recognition (CR) score

This note clarifies how the two-high-threshold (2HT) model relates to the corrected recognition (CR) score. We first outline the conditions under which CR corresponds to the 2HT detection parameter, then show why this equivalence breaks in the present asymmetric design, and finally explain how the 2HT model resolves these issues.

When CR corresponds to the 2HT detection parameter

The 2HT model provides a cognitive account of old–new recognition decisions (Snodgrass & Corwin, 1988). The model assumes that to evaluate a test item, the cognitive system transitions among mutually exclusive latent states of memory-based detection and guessing. Recognition is conceived as a discrete-state process with two detection states: an *old-detection state* ($D^{(\text{old})}$), entered when the item provides sufficient evidence of a memory match, and a *new-detection state* ($D^{(\text{new})}$), entered when the item provides positive evidence of novelty, i.e., evidence that no memory trace is present. When neither detection threshold is reached, the system enters a *guessing state* (g), and the old–new judgment is governed by guessing. This structure maps naturally onto the Multinomial Processing Tree (MPT) framework (Erdfelder et al., 2009), where latent cognitive states resemble a branching tree leading to observable old-new responses.

The transitions among latent processes generate observable responses. Hits arise either (i) when the old-detection state is reached ($D^{(\text{old})}$), or (ii) when the old-detection state is *not* reached ($1 - D^{(\text{old})}$) and leads to guessing "old" (g). These two possibilities give:

$$\Pr(\text{H}) = D^{(\text{old})} + \left(1 - D^{(\text{old})}\right) g.$$

False alarms (FAs) occur only when the new-detection state is *not* reached ($1 - D^{(\text{new})}$) and involves guessing "old" (g). Because both events must co-occur, their probabilities multiply:

$$\Pr(\text{FA}) = \left(1 - D^{(\text{new})}\right) g.$$

Under the standard identifiability assumption that both detection processes are governed by the same parameter, $D^{(\text{old})} \equiv D^{(\text{new})} \equiv D$ [1] the CR score ($\text{CR} = \Pr(\text{H}) - \Pr(\text{FA})$) simplifies to $\text{CR} \equiv D$. Therefore, CR can be interpreted as a bias-corrected measure of detection – implicitly adopting the assumptions of the 2HT model.

This interpretation requires that hit and false-alarm rates reflect the same underlying detection and guessing processes (i.e., matched old–new pairs and shared guessing). Under these conditions, CR differences map directly onto detection differences:

$$\begin{aligned}
\text{CR}_A - \text{CR}_B &= \left[\Pr(\text{H})_A - \Pr(\text{FA})_A\right] - \left[\Pr(\text{H})_B - \Pr(\text{FA})_B\right] \\
&\equiv \left[D_A^{(\text{old})} + (1 - D_A^{(\text{old})})g \;-\; (1 - D_A^{(\text{new})})g\right] - \left[D_B^{(\text{old})} + (1 - D_B^{(\text{old})})g \;-\; (1 - D_B^{(\text{new})})g\right] \\
&\equiv D_A - D_B, \qquad \text{given the identifiability assumption } D_k^{(\text{old})} \equiv D_k^{(\text{new})}.
\end{aligned}$$

[1] Beyond resolving the identifiability issue that arises when three latent parameters must be estimated from only two observable quantities (hit and false-alarm rates), this assumption also captures a standard theoretical view in the 2HT framework: the same underlying memory evidence is assumed to support both the detection of old items and the detection of new items. That is, the presence of a memory trace triggers an *old detection state*, whereas clear evidence of its absence triggers a *new detection state*, with both processes governed by the same detection parameter D.

Why CR fails in the present asymmetric design

The present design, however, does not satisfy the standard one-to-one correspondence between hits and false alarms. Targets are phase-specific (pre-, during-, post-conditioning), whereas lures are category-level (animals vs. tools), so each false-alarm rate is paired with three hit rates. This 3-to-1 asymmetry breaks the conditions under which CR reflects detection.

To illustrate the consequences of this asymmetry, we examine the comparison between conditions A1 and B1 under category-specific false alarms, analogous to our comparison of interest (i.e., the CS+ vs. CS− difference within each encoding phase):

$$
\begin{aligned}
\mathrm{CR}_{A1} - \mathrm{CR}_{B1} &= \left[\Pr(\mathrm{H})_{A1} - \Pr(\mathrm{FA})_{A}\right] - \left[\Pr(\mathrm{H})_{B1} - \Pr(\mathrm{FA})_{B}\right] \\
&= \left[D_{A1}^{(\mathrm{old})} + (1 - D_{A1}^{(\mathrm{old})})g - (1 - D_{A}^{(\mathrm{new})})g\right] - \left[D_{B1}^{(\mathrm{old})} + (1 - D_{B1}^{(\mathrm{old})})g - (1 - D_{B}^{(\mathrm{new})})g\right].
\end{aligned}
$$

This expression simplifies to:

$$
\mathrm{CR}_{A1} - \mathrm{CR}_{B1} = (1 - g)\big(D_{A1}^{(\mathrm{old})} - D_{B1}^{(\mathrm{old})}\big) + g\big(D_{A}^{(\mathrm{new})} - D_{B}^{(\mathrm{new})}\big).
$$

Thus, correspondence to the underlying detection difference requires one of two restrictive assumptions: the absence of guessing ($g = 0$) or memory-based detection being scaled by an *unknown* factor $(1-g)$ together with the equality of the novelty-detection processes across categories ($D_{A}^{(\mathrm{new})} \equiv D_{B}^{(\mathrm{new})}$). The latter is theoretically implausible, as it implies identical novelty detection for distinct sets of memory traces. As a result, in the current design CR no longer cleanly indexes detection. Instead, it combines detection differences with unknown contributions from guessing and novelty detection, violating the assumptions that give CR its usual cognitive interpretation.

How the 2HT model resolves the asymmetry

The full 2HT model, implemented as a multinomial processing tree, accommodates the 3-to-1 asymmetry. In our formulation, false alarms arise when the system fails to detect a match at *any* encoding phase, which is expressed by the functional relation:

$$
D^{(\mathrm{new})} \equiv f\left(D_{1}^{(\mathrm{old})}, D_{2}^{(\mathrm{old})}, D_{3}^{(\mathrm{old})}\right) = D_{1}^{(\mathrm{old})} \cdot D_{2}^{(\mathrm{old})} \cdot D_{3}^{(\mathrm{old})}.
$$

This captures the idea that detecting a *new* item requires successful non-match detection across all encoding phases – so any weak phase naturally increases false alarms. Crucially, false alarms are tied to the same memory parameters that generate hits. Consequently, detection and guessing remain identifiable, phase-specific effects are preserved, and false alarms contribute meaningful information.

Overall, these derivations indicate that CR is as a valid detection measure only when hit–FA pairings are matched – a condition not satisfied in the present design. Consequently, CR cannot be interpreted as a meaningful index of memory performance in this context. By contrast, the 2HT model in its full MPT specification provides a coherent alternative that respects the structure of the design and yields interpretable estimates of memory-based detection as stipulated by its theoretical background.

Appendix B: Treatment of confidence ratings and extensions of the 2HT model

Extending the 2HT model to incorporate confidence

In the standard 2HT model, an old/new decision is first made via detection or guessing. Confidence is assigned afterward, conditional on the latent state, so it can be modeled without changing the decision structure.

One simple extension assumes that high-confidence responses ("definitely") arise from the detection state and low-confidence responses ("maybe") from the guessing state. For old items:

$$\begin{aligned}
\Pr(DO \mid \text{old}) &= D^{(\text{old})}, \\
\Pr(MO \mid \text{old}) &= (1 - D^{(\text{old})})\, g, \\
\Pr(DN \mid \text{old}) &= 0, \\
\Pr(MN \mid \text{old}) &= (1 - D^{(\text{old})})(1 - g).
\end{aligned}$$

and for new items:

$$\begin{aligned}
\Pr(DN \mid \text{new}) &= D^{(\text{new})}, \\
\Pr(MN \mid \text{new}) &= (1 - D^{(\text{new})})(1 - g), \\
\Pr(DO \mid \text{new}) &= 0, \\
\Pr(MO \mid \text{new}) &= (1 - D^{(\text{new})})\, g.
\end{aligned}$$

This specification, however, imposes the unrealistic restriction that participants never produce "definitely new" responses to old items or "definitely old" responses to new items.

A more flexible alternative allows both detection and guessing states to generate high- and low-confidence responses, with detection yielding high confidence more often than guessing ($c_D > c_G$). This reflects the assumption that confidence is a post-decisional refinement rather than part of the detection mechanism itself. For old items:

$$\begin{aligned}
\Pr(DO \mid \text{old}) &= D^{(\text{old})} c_D + (1 - D^{(\text{old})})\, g\, c_G, \\
\Pr(MO \mid \text{old}) &= D^{(\text{old})}(1 - c_D) + (1 - D^{(\text{old})})\, g(1 - c_G), \\
\Pr(DN \mid \text{old}) &= (1 - D^{(\text{old})})(1 - g)\, c_G, \\
\Pr(MN \mid \text{old}) &= (1 - D^{(\text{old})})(1 - g)(1 - c_G),
\end{aligned}$$

and similarly for new items:

$$\begin{aligned}
\Pr(DO \mid \text{new}) &= D^{(\text{new})} c_D + (1 - D^{(\text{new})})(1 - g)\, c_G, \\
\Pr(MO \mid \text{new}) &= D^{(\text{new})}(1 - c_D) + (1 - D^{(\text{new})})(1 - g)(1 - c_G), \\
\Pr(DN \mid \text{new}) &= (1 - D^{(\text{new})})\, g\, c_G, \\
\Pr(MN \mid \text{new}) &= (1 - D^{(\text{new})})\, g(1 - c_G).
\end{aligned}$$

Reduction to the baseline 2HT model under binarization

Because confidence is assigned after the decision, high- and low-confidence responses from each state sum to the same underlying probabilities. Collapsing across confidence therefore recovers the standard 2HT model. For example:

$$\begin{aligned}
Pr(O \mid \text{old}) &= Pr(DO \mid \text{old}) + Pr(MO \mid \text{old}) \\
&= \left[D^{(\text{old})} c_D + (1 - D^{(\text{old})})\, g\, c_G\right] + \left[D^{(\text{old})}(1 - c_D) + (1 - D^{(\text{old})})\, g(1 - c_G)\right] \\
&= D^{(\text{old})}\left[c_D + (1 - c_D)\right] + (1 - D^{(\text{old})})\, g\, \left[c_G + (1 - c_G)\right] \\
&= D^{(\text{old})} + (1 - D^{(\text{old})})\, g.
\end{aligned}$$

Thus, adding confidence does not alter old/new response probabilities.
